# Income inequality and non-communicable disease mortality and morbidity in Brazil States: a longitudinal analysis 2002-2017

**DOI:** 10.1016/j.lana.2021.100042

**Published:** 2021-08-17

**Authors:** Renato Simões Gaspar, Ludovico Rossi, Thomas Hone, André Zuanazzi Dornelles

**Affiliations:** 1 Institute for Cardiovascular and Metabolic Research, School of Biological Sciences, University of Reading, Reading, UK; 2 Laboratory of Vascular Biology, Heart Institute (InCor), University of Sao Paulo School of Medicine, Sao Paulo, Brazil; 3 Department of Economics, CUNEF (Colegio Universitario de Estudios Financieros), Madrid, Spain; 4 Public Health Policy Evaluation Unit, School of Public Health, Imperial College London; 5 School of Biological Sciences, University of Reading, Reading, UK

## Abstract

**Background:**

Income inequality can negatively affect population health by increasing social stress and conflict, and reducing trust, public goods and healthcare access. However there is limited evidence from low and middle-income countries (LMICs) with high levels of inequality. This study investigates the association between income inequality, morbimortality and risk factors of non-communicable diseases (NCDs) in 26 Brazilian states from 2002 to 2017.

**Methods:**

Data was acquired for men and women from the Global Health Data Exchange, the Brazilian Institute of Geography and Statistics, and the Brazilian Ministry of Health, totalling 416 state-year observations. Disability-adjusted life years (DALYs) and risk factors of NCDs were the dependent variables. Gini Index was the main independent variable. Multivariate linear panel regressions were performed, controlling for state and time fixed effects, gross domestic product per capita, population ageing, poverty and access to healthcare.

**Findings:**

A 1% increase in the Gini Index was associated with increases in alcohol abuse (of 923•4 DALYs per 100,000 people, 95%CI 217•6 to 1629•0) and diabetes mellitus morbidity (of 893•3 DALYs per 100,000 people, 95%CI 127•7 to 1659•0), and decreases in morbidity from attention disorder (of -4•0 DALYs per 100,000 people, 95%CI -7•4 to -0•5) and autism spectrum (of -2•4 DALYs per 100,000 people, 95%CI -4•3 to -0•5). These associations were greater for men, further supported by associations with alcohol use as a risk factor.

**Interpretation:**

This study provides evidence from a highly unequal LMIC, Brazil, of negative associations between income inequality and NCDs, and the importance of addressing wider social determinants of health.

**Funding:**

This study was financed in part by the Coordenação de Aperfeiçoamento de Pessoal de Nível Superior - Brazil (CAPES) - Finance Code 001 as a Brazilian CAPES scholarship to AZD and by the São Paulo Research Foundation (FAPESP), grant 2020/15944-8 to RSG.

**Research in context**

**Evidence before this study**

We searched MEDLINE and Google Scholar using broad keywords such as “income inequality” or “Gini Index” and “disease”, “mortality”, “morbidity” or “health”. Results from Google Scholar and MEDLINE varied between hundreds to thousands of studies depending on the term used. Review papers tended to agree that income inequality does affect population health. Gini Index was linked with specific disease outcomes in either specific countries or, less frequently, a panel of countries. Amongst the different diseases assessed, cardiovascular diseases were the most common. Studies conducted in the US or Brazil generally showed a statistically significant association between income inequality and specific disease outcomes. Few studies assessed this correlation with risk factors for non-communicable diseases (NCDs) and, to the best of our knowledge, none assessed the association between income inequality and the burden of morbidity from NCDs. A minority of papers used fixed-effects models and stratified the population in subgroups of men and women, showing no association between income inequality and population health. There was no work thoroughly assessing a wide panel of NCDs, accounting for a wide range of risk factors, confounding factors, and fixed-effects.

**Added value of this study**

This is the first study to thoroughly assess the effects of income inequality for over 60 NCDs outcomes, using the most granular data available from the GBD database. The use of fixed-effects econometric models, controlling for income, ageing, poverty and access to healthcare, allowed a more comprehensive understanding of the link between income inequality and population health in a low and middle-income country (LMIC), namely Brazil. The analysis was performed on Brazil because 1) studies on other LMICs are limited, 2) it provides high-quality subnational data for a relatively long time period, and 3) it presents staggering differences across states in terms of income and NCDs outcomes. There was no association between income inequality and the overall burden of all NCDs, however income inequality was associated with higher burdens from diabetes- and alcohol-related diseases, and lower burdens of mental health conditions - particularly for men. For men, income inequality was also associated with increases in the SEV of alcohol use - partially explaining the identified associations for alcohol-related illnesses.

**Implications of all the available evidence**

This study provides a detailed analysis of the association between income inequality and more than 60 outcomes of NCDs in Brazil. The nuances of how income inequality is associated with NCDs suggest important sex- and disease-specific effects. This study contributes to the growing evidence of the negative associations of inequality and health and that these negative realtionships are found in highly unequal LMICs such as Brazil. These findings draw attention to the importance of tackling wider social determinants of health and the synergistic benefits from tackling inequalities for the social developmental goal (SDG) 10 (inequalities) and SDG 3 (health). This information is of particular interest to public policymakers since it elucidates novel and improved ways in which income inequality may be detrimental to population health.

Alt-text: Unlabelled box

## 1 Introduction

Reducing inequalities within and between countries is the 10^th^ Sustainable Development Goal of the United Nations. The COVID-19 pandemic has substantially affected inequalities with the poorest and most vulnerable populations negatively affected the most [1]. Evidence links higher income inequality to poorer population health [2] with rising income inequality associated with increase in the burden of non-communicable diseases (NCDs) in high-income countries [3,4]. There is little knowledge from low- and middle-income countries (LMICs) on the relationship between income inequalities and population health, which is vital as LMICs have experienced rapid economic growth in recent years and often have weak health and social protection systems.

Increases in income have a well-established beneficial effect on population health [5]. This association, however, is not homogenous as income increases for the poor deliver greater health benefits than increases for the rich [5]. Inequalities in income also affect population health both directly and indirectly. The psychosocial interpretation suggests that the perception of others within a social and income hierarchy can lead to stress, with large differences in this hierarchy leading to chronic stress and poorer population health [6]. Inequality can affect an individual's ability to afford healthcare services [7] and can also affect the quality these services (e.g. creating obstacles to public health care in marginal communities, or exposing communities to environmental and sanitary risks) [7]. Income inequality may increase the frailty of the social fabric and reduce mutual trust [8], resulting in reductions in public goods that protect population health [9,10]. Increase in inequality foster increased hostility, violence, racism and other forms of discrimination. It has also been proposed that inequality can reduce the speed of innovation diffusion across income groups [11], meaning the poor are less likely to access new and beneficial medical innovations. Therefore, multiple causal mechanisms in the individual, social and structural levels link income inequality to poorer population health.

Previous evidence corroborates the association between specific-cause NCDs mortality and income inequality [12]. Moreover, the association between income inequality and specific health outcomes increases with inequality [13]. However, this association is often inconsistent in studies that use data from multiple countries, as often social, cultural and political particularities that are not accounted for [14,15]. Indeed, several studies have often pooled men and women together [16,17] or performed cross-country studies without fully accounting for potential national differences [15,^18^,19]. The use of controls for unobserved time and geographical characteristics (i.e. fixed-effects) are a methodoligcal advancement for analysing health data. Generally, more robust fixed-effects studies showed little or no association between income inequality and health outcomes [15,18]. However, there is a lack of studies controlling for comprehensive contextual factors, such as medical assistance and public health coverage, as well as studies in LMICs.

Brazil is one of the most unequal countries in the world, including on income and health inequalities and, despite remarkable socioeconomic improvements in the last three decades, inequalities persist [20,21]. Improvements in socioeconomic factors have not occurred at the same time and scale across all the regions of Brazil. Richer households are concentrated in the South and Southeast regions and poorer families are concentrated in the North and Northeast regions, reflecting a North-South gradient in terms of human development and access to public services [22]. Moreover, improvements in life expectancy have been accompanied by higher morbidity and mortality burdens from NCDs such as diabetes and cancers [23]. However, the role of income inequality in affecting NCDs in Brazil is under-explored – like many LMICs.

We aimed to assess the association between income inequality, in the form of the Gini index, and mortality, morbidity and risk factors for NCDs in Brazil, from 2002 to 2017. An extensive set of disease subgroups were explored to better understand the nuances of the complex association between income inequality and population health.

## 2 Methods

### 2.1 Study design

This study was a panel regression analysis where the units of analysis were the states of Brazil (n=26). Panel regression models are appropriate for exploring assoications between geographical areas over time and account for the hiererachical (clustered) nature of the data (i.e. year observations per state).

### 2.2 Data sources

Data used in this study were collected from publicly available datasets: the Global Health Data Exchange (GHDx), the Institute for Health Metrics and Evaluation (IHME), the Brazilian Institute of Geography and Statistics (IBGE) and the Brazilian Ministry of Health. Data were acquired for the 26 states from 2002 to 2017, totalling 416 state-year observations. Supplementary Table 1 presents descriptive information about variables and data sources used.

### 2.3 Outcomes

The dependent variables were the rate per 100,000 habitants of disability-adjusted life years (DALYs rate) and the summary of exposure value (SEV) of risk factors. DALYs is the sum of years of life lost due to premature mortality (YLL, calculated for each cause-specific death relative to the normative standard life expectation at the age of death) and years lived with disability (YLD, calculated by the prevalence of each disease sequela by its disability weight). DALYs, in this sense, express years of healthy life lost and thus reveals a comprehensive understanding of fatal and non-fatal epidemiological burden of diseases. SEV is a measure of a population's exposure to a risk factor that takes into account the extent of exposure by risk level and the severity of that risk's contribution to disease burden. Each dependent variable was used independently, in different regression models.

Different NCDs, such as type I and type II diabetes, cardiovascular and pulmonary diseases, and distinct cancers were selected according to their clinical and epidemiological significance, based on previous literature [24]. In addition, we collected data for risk factors that met causal criteria for the NCDs analysed in this study, as described previously [25]. All dependent variables were collected for men and women separately. Supplementary Table 2 reports the full description of the metrics used in this study. Supplementary Table 3 presents the International Classification of Diseases (ICD) codes for NCDs used. Supplementary Table 4 reports the metadata of risk factors [25]. NCDs were categorized in the following subgroups, according to ICD codes: cardiovascular diseases, chronic respiratory diseases, diabetes and kidney diseases, mental disorders, neurological disorders, musculoskeletal disorders, substance use disorders, digestive diseases, gynaecological diseases and neoplasms. Specific diseases outcomes (e.g. type 2 diabetes, ischaemic stroke, etc) are presented for each NCDs subgroup. A previous report by the Global Burden of Disease Study 2016 (GBD 2016) attested the quality of data from Brazilian official sources, with mild levels of imprecision indicated by15% to 35% of deaths that were not well-certified in terms of ideal completeness, availability, and detail of mortality data [24].

The main independent variable was the state-level Gini Index of household income, which is the most widely used income inequality index. Higher Gini Index reflects higher income inequality, therefore a Gini Index of 0 corresponds to perfect income equality (i.e. all individuals have the same income), whereas a Gini Index of 1 corresponds to complete inequality (i.e. one person has all the income, while the remaining part of the population has no income). Data on relevant covariates were obtained to control for known contributors of population health, which were: gross domestic product (GDP) in R$ (Reais) per capita to account for the effects of income on population health [5]; the percentage of the population over 60 years of life to account for population ageing; the number of public hospital beds; number of medical doctors per 1,000; coverage of private healthcare and coverage of primary care [26] to account for access to healthcare; and Bolsa Família value in R$ (Reais) to control for transfers from the government to the poorest groups of the population [27] to account for poverty. There were no missing data for the state-year observations included in the regression models.

### 2.4 Modelling approach

Data were analysed through multivariate linear regressions with year and state fixed effects, performed using STATA 15 software. Models were fitted using health outcomes (DALYs per 100,000, YLLs per 100,000 or SEV of risk factors) as the dependent variable, the Gini Index as the main independent variable and a set of other control variables as confounders. Clustered standard errors at the state level were used to account for heteroskedasticity and autocorrelation [28]. Degrees of freedom were calculated according to Wooldridge [28]. State fixed-effects accounted for unobserved factors such as cultural, geographic and historical variables, which vary across states but are fixed over time. Likewise, year fixed-effects accounted for unobserved factors that vary across time but not across states. Fixed effects models are considered robust, suitable to analyse aggregated health data [29] and were estimated using Ordinary Least Square. The use of fixed-effects reduced the likelihood of multiplicity in the constructed models. The output of the model can be interpreted as the association between outcomes and Gini index.

The first multivariate linear regression model consisted of an unadjusted approach using the dependent variable, Gini index, GDP per capita and the time and location fixed effects. The unadjusted model is

$$healthoutcome_{it}=\alpha +\beta_{1}Gini_{it}+\beta_{2}lnGDP_{it}+L_{t}+T_{l}+\varepsilon_{it},$$

where is DALYs per 100,000, YLLs per 100,000 or SEV of risk factors for a given disease or disease group in a state *l* in year *t*: is the Gini Index of state *l* in year *t,* is the logarithm of GDP per capita. *L* are state fixed effect and *T* are time fixed effect.

To extend the analysis, confounding variables were added to the unadjusted model, as exposed above. Thus, the adjusted model is

$$healthoutcome_{it}=\alpha +\beta_{1}Gini_{it}+\beta_{2}lnGDP_{it}+\beta_{3}C_{it}+L_{t}+T_{l}+\varepsilon_{it},$$

where the *C_lt_* represented the cofounding variables that are the percentage of population over 60 years of age, the number of doctors per 1,000 habitants, the number of hospital beds per 1,000 habitants, the coverage of primary healthcare, the coverage of private healthcare and Bolsa Família transfer. The percentage of population living in urban areas and the percentage of white individuals within states were included in an additional model to test the robustness of our data.

### 2.5 Role of the funding source

This study was financed in part by the Coordenação de Aperfeiçoamento de Pessoal de Nível Superior - Brazil (CAPES) - Finance Code 001 as a Brazilian CAPES scholarship to AZD. The funding agecy had no role in the design, data collection, analysis or decision to publish. The corresponding author had full access to all the data in the study and had final responsibility to submit for publication.

## 3 Results

**Figure 1:**
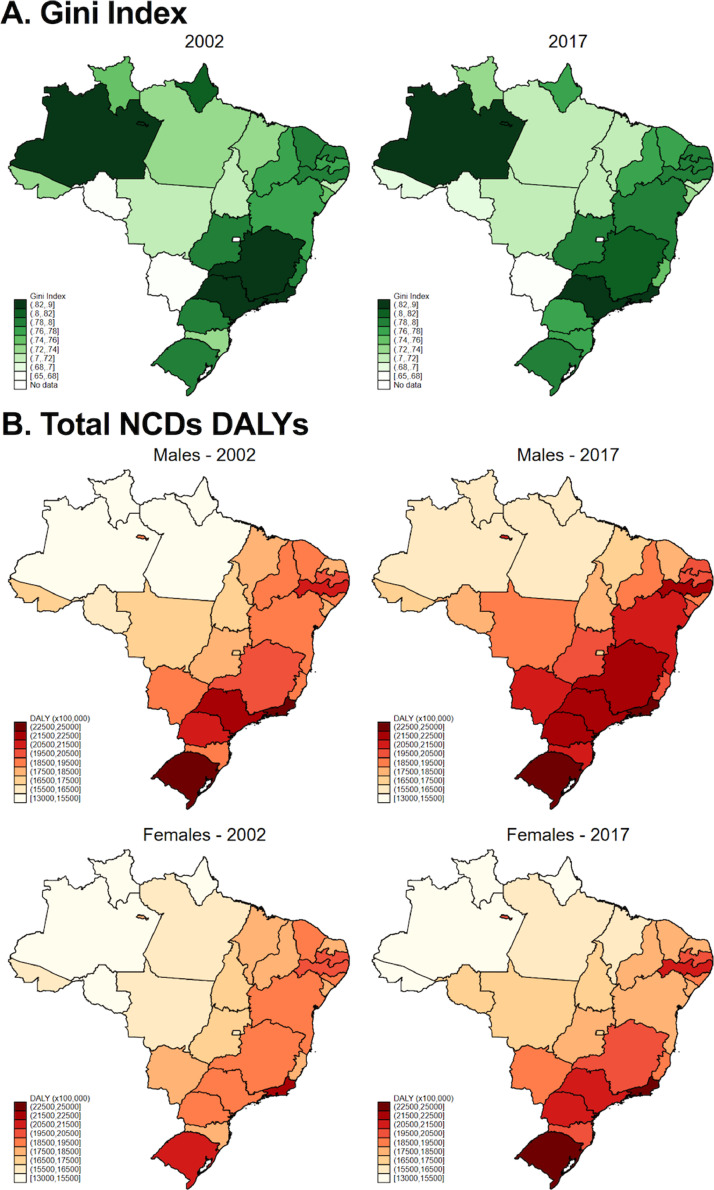
**Gini Index and DALYs rate of all NCDs in 2002 and 2017 in Brazil.** (A) Subnational Gini Index was collected from the Brazilian Institute of Geography and Statistics (IBGE). (B) Disability-adjusted life years (DALYs) for non-communicable diseases (NCDs) was collected for both men and women between 2002 and 2017. DALYs rate is experessed as DALYs per 100,000 people.

Income inequality measured by the Gini index decreased on the majority of states from 2002 to 2017, with a few exceptions, such as Santa Catarina in the South region (which increased from 0•74 in 2002 to 0•76 in 2017) and Rondônia in the North region (0•66 in 2002 and 0•69 in 2017 - Figure 1A and Supplementary Table 5). The Gini Index was positively correlated with *ln*GDP per capita (r = 0•531), suggesting that richer states tended to present higher levels of income inequality (Supplementary Table 6). In parallel, there was a visible increase in DALYs rate accountable to all NCDs in all states, for both men and women (Figure 1B and Supplementary Table 7), likely due to increased life expectancy (Supplementary Table 7).

**Figure 2:**
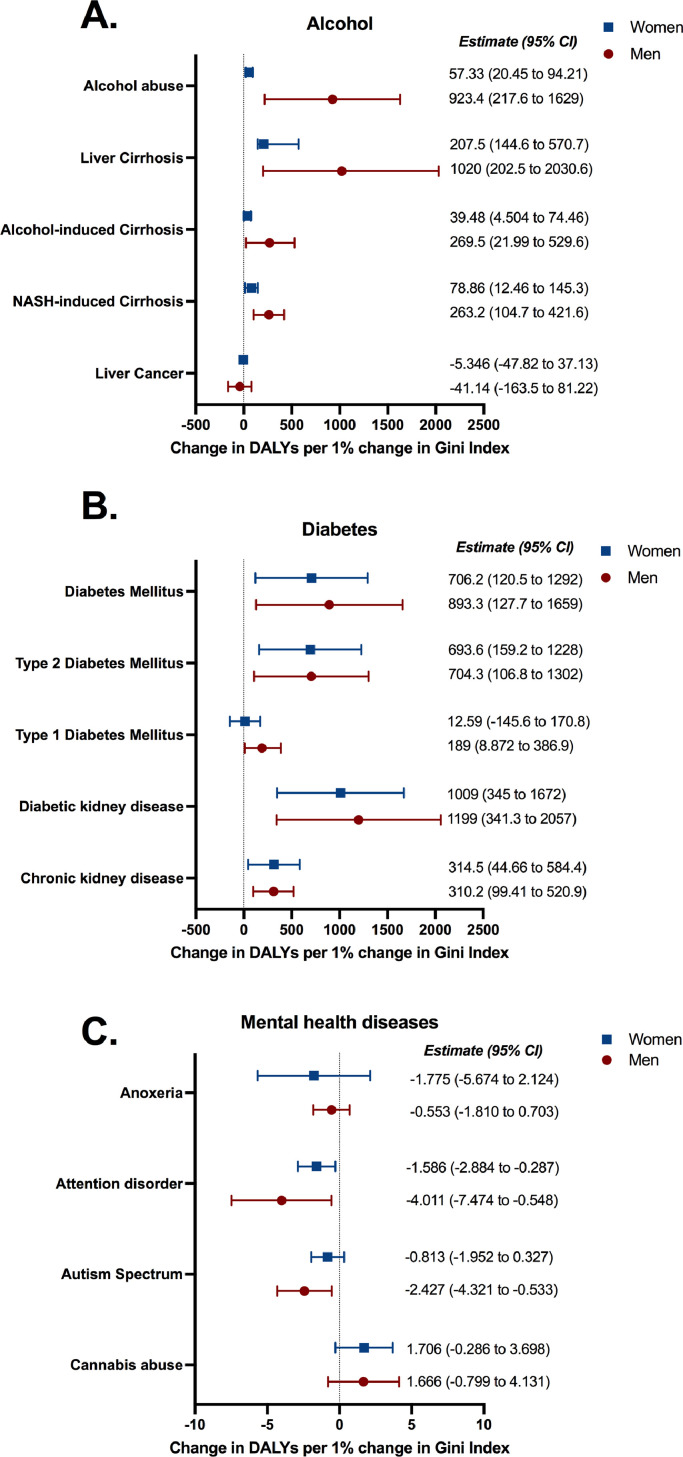
**Associations between Gini Index and DALYs rate for (A) alcohol-, (B) diabetes-, and (C) mental health-related diseases in men and women in Brazil.** The associations presented were only deemed statistically significant if present in both unadjusted and adjusted models, as per Supplementary Table 8. Variables included in the adjusted model: doctors per 1,000 habitants, hospital beds per 1,000 habitants, coverage of private healthcare, coverage of primary care, Bolsa Família transfer, percentage of the population over 60 years of age and state and year fixed effects. DALYs rate is experessed as DALYS per 100,000 people. Estimates and 95% confidence intervals (CI) are provided in the figure.

Regression analysis adjusting for GDP per capita, ageing, access to healthcare and poverty were performed for the most relevant NCDs in Brazil [24] and are shown in Supplementary Table 8. Only regression coefficients detected in both unadjusted and adjusted models were considered robust and are presented in main figures. No association was found between the overall burden of NCDs and Gini Index (Supplementary Table 8), consistent with observations in Figure 1. When analysing specific NCD subgroups, alcohol-, diabetes- or mental health-related illnesses were statistically significantly associated with income inequality (Figure 2). In adjusted regression models a 1 percentage point change in the Gini Index was associated with an increase in alcohol abuse (of 923•4 DALYs per 100,000 people, 95%CI 217•6 to 1629•0) and diabetes mellitus (of 893•3 DALYs per 100,000 people, 95%CI 127•7 to 1659•0). There were also positive associations for alcohol- and diabetes-related diseases, such as liver cirrhosis and diabetic kidney disease, respectively, for both men and women. Supplementary Figure 1 shows a linear correlation between DALYs of diabetes mellitus in men and Gini Index for the year of 2017 to provide a visual representation of the association observed using the regression models.

In contrast, there were negative associations between Gini Index and DALYs from some mental health diseases (Figure 2C). In adjusted regression models a 1% change in the Gini Index was associated with a decrease in attention disorder (of -4•0 DALYs per 100,000 people, 95%CI -7•4 to -0•5) and autism spectrum (of -2•4 DALYs per 100,000 people, 95%CI -4•3 to -0•5). However, the magnitude of these negative associations was considerably lower than the positive associations reported for alcohol- and diabetes-related diseases. Moreover, data gathered in Figure 2 show that the associations for alcohol-related and mental health diseases were more prominent in men, suggesting sex-specific effects. Results for all diseases assessed are presented in Supplementary Table 8. These findings were comparable when using YLLs instead of DALYs, suggesting that increased income inequality was associated with higher mortality of alcohol- and diabetes-related diseases (Supplementary Figure 2 and Supplementary Table 9). In contrast, several mental health diseases, such as autism and cannabis abuse, were not considered causes of death, implicating that income inequality was associated only with the morbidity (YLDs) of mental health diseases. Moreover, a robustness test was performed by the addition of urbanicity and race/colour as covariates within the extended model (Supplementary Figure 3). This analysis revealed equivalent associations to the original extended model, suggesting that the associations reported in our study are robust.

**Figure 3:**
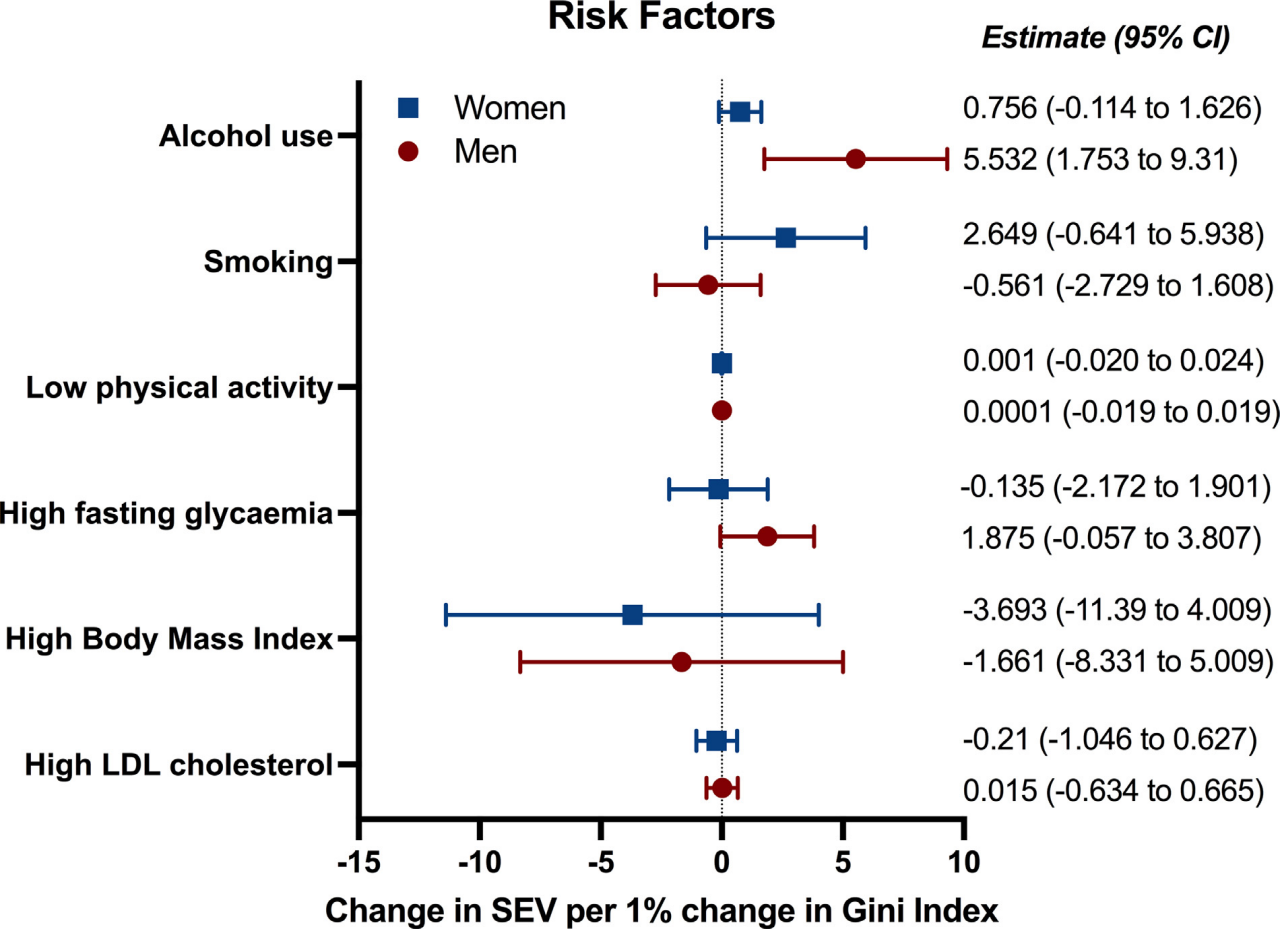
**Associations between Gini Index and summary exposure value (SEV) of risk factors for non-communicable diseases (NCDs) for men and women in Brazil.** The associations presented were only deemed statistically significant if present in both unadjusted and adjusted models, as per Supplementary Table 9. Variables included in the adjusted model: doctors per 1,000 habitants, hospital beds per 1,000 habitants, coverage of private healthcare, coverage of primary care, Bolsa Família transfer, percentage of the population over 60 years of age and state and year fixed effects. Estimates and 95% confidence intervals (CI) are provided in the figure.

The association between risk factors for NCDs and changes in income inequality was also explored (Figure 3) [25]. For men, a 1% change in the Gini Index was associated with an increase in alcohol use (of 5•5 SEV per 100,000 people, 95%CI 1•7 to 9•3). Regression results for all risk factors assessed are presented in Supplementary Table 10. The associations found for other risk factors (e.g. diet low in vegetables) were not considered robust, since these were only present in the adjusted model. Altogether, it is likely that associations found for DALYs of alcohol-related diseases in men are partially attributed to rises in alcohol use associated with greater income inequality.

## 4 Discussion

This study provides a detailed analysis of the association between income inequality and more than 60 outcomes of NCDs in Brazil. There was no association between income inequality and the overall burden of all NCDs, however income inequality was associated with higher burdens from diabetes- and alcohol-related diseases, and lower burdens from mental health conditions - particularly for men. For men, income inequality was also associated with increases in the SEV of alcohol use - partially explaining the identified associations for alcohol-related illnesses. Therefore, the nuances of how income inequality is associated with NCDs suggest important sex- and disease-specific effects.

Previous studies have shown correlations between Gini Index and specific NCDs outcomes, such as stroke in Brazil [12], coronary heart disease in the US [13] and diabetes mortality in HICs [30]. There is also literature showing no association between income inequality and depression in LMICs [31] and cardiovascular diseases in HICs [15]. The time period, type of regression and control variables, however, differed greatly amongst the abovementioned studies. Despite no association between all-cause DALYs rate and income inequality in our study, which is similar to other reports [15,^17^,31], there were statistically significant associations found for specific disease groups. It is possible that by pooling data from different Brazilian states our model could not capture differences in highly aggregated variables, such as all-cause DALYs. In addition, we report a negative association between DALYs rate of some mental health diseases, namely autism spectrum and attention disorder and income inequality. It is possible that some NCDs are positively affected by income inequality while other are negatively affected, thus resulting in an overall lack of association. Therefore, it is advisable to consider more granular outcomes of morbi-mortality of NCDs when studying social determinants of health. This is in accordance with a recent review suggesting that the relationship between income inequality and health needs to be examined in more detail and is likely more complex than previously thought [7].

The finding of an association between increases in income inequality and lower mental health conditions is contrary to much of the literature [32]. There are multiple possible exaplanations for this finding, including the possibility that specific mental health conditions are differentially associated with income inequality. For instance, the prevalence of autism spectrum disorder is higher in children born in richer neighborhoods [33], an effect likely due to diagnostic bias and access to healthcare services. In parallel, higher income inequality was shown to increase the risk of depression, but not anxiety, in a cross-sectional study in the city of Sao Paulo, Brazil [34], suggesting that different mental health disorders may be differently affected by income inequality. Contrasting results between the abovementioned reports and our data may be due to the use of different controls and statistical methods. In spite of a statistically significant negative association between some mental health disorders and income inequality in our study, it must be noted that the magnitude of this association is ∼1000-fold lower than that found for alcohol- and diabetes-related diseases.

Several theories have attempted to explain the association reported between income inequality and health. Two of the most widely accepted are the psychosocial [8] and neo-materialist [9], [10] theories. The first poses that perception of a lower place in the social hierarchy generates stress that ultimately increases disease risk so that greater social gradient leads to higher levels of stress, whereas the latter suggests that poorer individuals have less access to medical assistance than richer ones and therefore would display worse health outcomes. One could argue that alcohol consumption is a stress-related behaviour, while diabetes-related diseases require access to more specific medical assistance. Indeed, previous studies have found similar associations between state-level income inequality and alcohol-related problems [35] and diabetes [36]. The reality, however, is more likely a mixture of both interpretations [7] and other sex- and disease-specific factors. In our analysis, we used time and location fixed effects, which accounted for slow changes within society that are hard to reliably measure, thus considering the psychosocial factors involved. Conversely, we also employed control variables for medical assistance, poverty and ageing as surrogates for neo-materialism. Yet after adjusting for potential confounders, we found associations between income inequality and diabetes-, alcohol- and mental health-related illnesses, evidencing the need of more refined theories to explain how income inequality may affect population health, including the consideration of sex- and disease-specific factors. These should be explored in other countries through the use of more granular data and through the comparison of the effect of income inequality for men and women.

**Figure 4:**
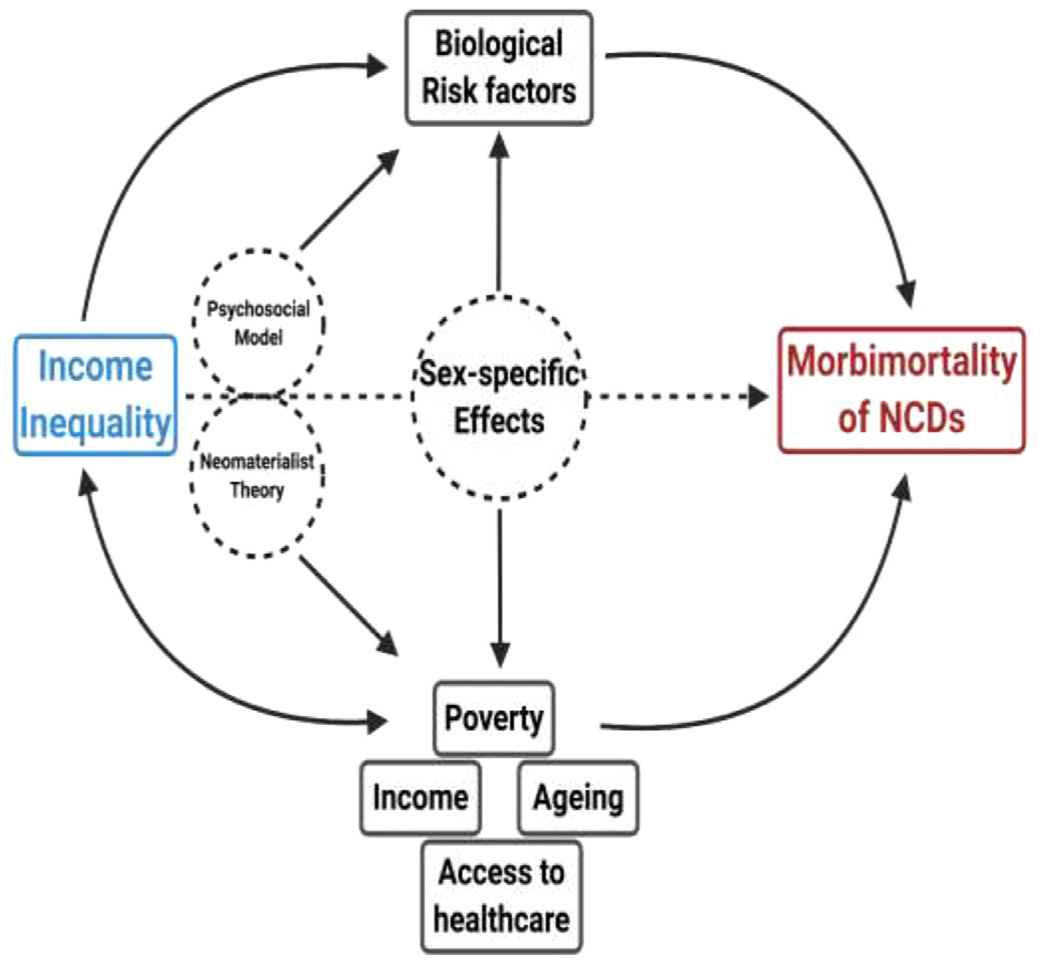
**Proposed model of how income inequality may be associated with morbimortality of non-communicable diseases (NCDs).** Income inequality may affect population health through several pathways: higher frequency of stress behaviour as a direct consequence of income inequality (e.g. alcohol use in men), influence of other social factors that contribute to disease risk (e.g. poverty, income) and sex-specific effects that may affect particular diseases (e.g. increased cardiovascular outcomes due to diabetes in men) or social behaviour (e.g. lower healthcare services utilization by men).

We report a considerable sex-specific effect, in which men seem to be more affected by income inequality when compared to women. For instance, the DALYs rate of alcohol abuse in men was 16 times more affected by income inequality than the DALYs rate of alcohol abuse in women (923 days-change for men *vs* 57 days-change for women per 1% change in Gini Index, Figure 2A). In line with our findings, in the US, Pinkhasov et al [37] provided evidence that men were more likely to be heavy alcohol drinkers and less likely to utilize health care services when compared to women. Moreover, a large retrospective study in Canada has shown that diabetic men entered the high-risk category for cardiovascular event 7 years earlier than diabetic women [38], suggesting that sex-specific characteristics, either biological or social, result in aggravated cardiovascular outcomes for men. Altogether, income inequality may affect population health through several pathways: higher frequency of stress behaviour as a consequence of income inequality [35] (e.g. alcohol use in men), influence of other social factors that contribute to disease risk [36] (e.g. poverty) as well as sex-specific effects that may influence particular diseases [38] (e.g. increased cardiovascular outcomes due to diabetes in men) or social behaviour [37] (e.g. lower healthcare services utilization by men). This workflow has been summarized in Figure 4.

The association of income inequality with different NCDs also raises the debate on how the reduction of income inequality can synergistically tackle two Sustainable Development Goals, namely SDG 10 (inequalities) and SDG 3 (health). A better comprehension of such links is relevant to healthcare planning and resource allocation within the Brazilian Unified Health System (*Sistema Único de Saúde*, SUS) since suboptimal resource allocation within SUS is a matter of constant debate [39,40]. Notably, associations found between particular risk factors (e.g., diet high in red meat) and income inequality that were not extended to their respective NCD (e.g., colon and rectum cancer [41]) can be of important strategical benefit for healthcare planning and efficiency from a preventive perspective. In addition, improvements to the Healthcare Access and Quality (HAQ) Index (which measures the performance to restrain preventable deaths) slowed down nationally between 2000 and 2016, whilst subnational disparities slightly increased [42]. Such findings suggest that improved access to healthcare was not necessarily followed by better quality (particularly for NCDs) and can partially explain why coverage of primary healthcare was correlated with DALYs from NCDs (Supplementary Table 6). Finally, income-transfer programmes such as *Bolsa Família*, which has been shown to reduce income inequality [43,44] in support with our findings (negatively correlated with Gini Index and DALYs from NCDs – Supplementary Table 6), could prove benefits in mitigating the health impacts of income inequality.

### 4.1 Study limitations

Our study has limitations that should be taken into account. Firstly, the use of secondary databases commonly raises issues around data quality and processing. Mortality measurements were under-represented in Brazil throughout the time of analysis, especially in poorer states [24]. However, the data used was still considered of high quality despite some under-reporting [24]. Data from IHME were smoothed by IHME using an algorithm developed by this source [24], which could potentially mask subtle changes over time. To minimize these potential limitations, we used state and time fixed effects to decrease the likelihood of unobserved changes in mortality reporting being associated with changes in income inequality [45].

Secondly, despite using a longer period of analysis than similar studies, it is possible that this approach did not fully capture chronic additive effects of income inequality. This is especially relevant when analysing risk factors. The relation between income inequality and risk factors may require a time lag to be measurable on their actual impact to morbidity or mortality of diseases. Therefore, a time lag analysis should be performed when a longer period of analysis becomes available to fully acknowledge the historical cumulative effects of income inequality in Brazil and other LMICs. Thirdly, the unit of analysis was the state and as large geographical areas this obscures more detailed individual-level associations between inequality and health. Effect estimates in this study reflect averages on effects in poor and wealthier individuals. Fourthly, the Brazilian States assessed showed high level of income inequality and a relatively narrow range in Gini index values (varying from 0.66 to 0.89). Thus, interpretations of the results beyond similar settings of income inequality should be taken with care.

Finally, causal interpretations cannot be made. Despite the use of both fixed effects and control variables in our adjusted model, which reduce the influence of unobserved variables, the presence of other time-varying confounding factors cannot be entirely excluded. Thus, there is a need to further explore the causal effect of income inequality in specific contexts, populations, timeframes, and respective NCDs outcomes.

## 5 Conclusion

Income inequality is associated with increases in diabetes- and alcohol-related diseases and very small reductions in mental health-related conditions in the Brazil, particularly for men. This study contributes to the growing evidence of the negative associations between inequality and health and that these negative realtionships are found in highly-unequal LMICs such as Brazil. The findings draw attention to the importance of tackling the wider social determinants of health and the synergistic benefits from tackling inequalities for SDG 10 (inequalities) and SDG 3 (health).

## Contributors

All authors made substantial contributions to the content and writing of this paper. RSG conceived the study and wrote the first draft. RSG and AZD designed the study and collected data. LR performed data analysis and data interpretation. TH provided inputs on statistical methods and interpretation. All authors made substantial intellectual contributions to several drafts.

## Declaration of interests

We declared no competing interests.

## Data sharing statement

Data used in this manuscript are freely available from the sources mentioned and have been compiled as a spreadsheet uploaded as an e-component.

## Appendix Supplementary materials

Image, application 1

Image, application 1

Image, application 1

Image, application 1

Image, application 1

Image, application 1
