# Supplementary material for: Income inequality and non-communicable disease mortality and morbidity in Brazil States: a longitudinal analysis 2002-2017
Source: Lancet Reg Health Am. 2021 Aug 17;2:100042. doi: 10.1016/j.lana.2021.100042 (PMC9904117; doi:10.1016/j.lana.2021.100042)
Supplement: Supplementary file 1 [file mmc1.docx]

**Supplement to:** Gaspar RS*, Rossi L, Hone T, Dornelles AZ. **Income inequality and non-communicable disease mortality and morbidity in Brazil States: a longitudinal analysis 2002-2017**

*Corresponding author:

Renato Simões Gaspar

E-mail: renatosgaspar@gmail.com

Tel: +44 11 8378 7047

## Supplementary Table 1: Descriptive information on variables and data sources explored across 26 Brazilian states from 2002 to 2017.

| **Variable name** | **Unit** | **Source** | **Date last accessed** |
| --- | --- | --- | --- |
| **Socioeconomic indicators and care services** | | |  |
| GDP per capita | Reais (R$) | IBGE. Gross domestic product at current prices, taxes, net of subsidies, on products at current prices and gross value added at current prices, total and by economic activity, and respective shares – Reference year of 2010. Available at: https://sidra.ibge.gov.br/tabela/5938 | 14^th^ July 2021 |
|  | NA | IBGE/Gini index of gross domestic product at current prices and gross value added at current prices – Reference year of 2010. Available at: https://sidra.ibge.gov.br/tabela/5939 | 14^th^ July 2021 |
| Hospital beds | Beds per 1,000 inhabitants | Brazilian Ministry of Health /SAS – National Registry of Health Facilities (CNES). Available at: http://tabnet.datasus.gov.br/cgi/deftohtm.exe?cnes/cnv/leiintbr.def | 18^th^ July 2021 |
| Doctors rate | Doctors per 1,000 inhabitants | Brazilian Ministry of Health/SGTES/DEGERTS/CONPROF – Professional Councils. Available at: http://tabnet.datasus.gov.br/cgi/deftohtm.exe?cnes/cnv/prid02br.def | 14^th^ July 2021 |
| Coverage of private healthcare | % of people | Brazilian Ministry of Health – National Health Agency. Available at: http://www.ans.gov.br/anstabnet/cgi-bin/dh?dados/tabnet_tx.def | 13^th^ July 2021 |
| Coverage of primary care | % of people | Brazilian Ministry of Health – National Health Agency. Available at: https://bit.ly/2SQBSWf | 14^th^ July 2021 |
| Bolsa Família | Reais (R$) | Brazilian Ministry of Social Development. Available at: https://aplicacoes.mds.gov.br/sagi/vis/data3/data-explorer.php | 23^rd^ Jun 2021 |
| Prevalence of population >60 years old | % of people | Global Health Data Exchange/GBD Results Tool. Available at: http://ghdx.healthdata.org/gbd-results-tool | 14^th^ july 2021 |
| **Health outcomes and risks** | | |  |
| Disability-Adjusted Life Years (DALYs) | Years | Global Health Data Exchange/GBD Results Tool. Available at: http://ghdx.healthdata.org/gbd-results-tool | 14^th^ July |
| Life expectancy and Healthy life expectancy (HALE) | Years | Global Health Data Exchange/GBD Results Tool. Available at: http://ghdx.healthdata.org/gbd-results-tool | 14^th^ July |
| Summary Exposure value (SEV), risk factors | Risk-adjusted prevalence, from 0 to 1 | Global Health Data Exchange/GBD Results Tool. Available at: http://ghdx.healthdata.org/gbd-results-tool | 14^th^ July |

^a^Gini Index was not measured in 2002 and 2017; ^b^Hospital beds were not available in 2004.

## Supplementary Table 2: Glossary of metrics.

| **Metric** | **Definition** |
| --- | --- |
| Gini Index | Gini index measures the extent to which the distribution of income (or, in some cases, consumption expenditure) among individuals or households within an economy deviates from a perfectly equal distribution. A Lorenz curve plots the cumulative percentages of total income received against the cumulative number of recipients, starting with the poorest individual or household. The Gini index measures the area between the Lorenz curve and a hypothetical line of absolute equality, expressed as a percentage of the maximum area under the line. Thus, a Gini index of 0 represents perfect equality, while an index of 100 implies perfect inequality. |
| GDP per capita | GDP per capita is gross domestic product divided by midyear population. GDP is the sum of gross value added by all resident producers in the economy plus any product taxes and minus any subsidies not included in the value of the products. It is calculated without making deductions for depreciation of fabricated assets or for depletion and degradation of natural resources. |
| Year of Life Lost (YLL) | Years of life lost due to premature mortality. YLLs are the multiplication of deaths and a standard life expectancy at the age of death. The standard life expectancy is derived from a life table that contains the lowest observed mortality rate at each age that has been observed in any population greater than 5 million. |
| Years Lived with Disability (YLD) | Years lived with any short-term or long-term health loss weighted for severity by the disability weights. |
| Disability-Adjusted Life Years (DALYs) | The sum of years lost due to premature death (YLLs) and years lived with disability (YLDs). DALYs are also defined as years of healthy life lost. |
| Life expectancy | The number of years a person is expected to live at a given age assuming he or she will experience the age-specific mortality rate observed in a given year throughout his or her lifetime. For GBD, the life expectancy associated with an age group (e.g., 50- to 54-year-olds) is life expectancy at the starting year of the age group. |
| Summary Exposure value (SEV) | A measure of a population’s exposure to a risk factor that takes into account the extent of exposure by risk level and the severity of that risk’s contribution to disease burden. SEV takes the value zero when no excess risk for a population exists and the value one when the total population is at the highest level of risk; we report SEV on a scale from 0% to 100% to emphasize that it is risk-weighted prevalence. |

## Supplementary Table 3: International Classification of Diseases (ICD) codes and hierarchy for non-communicable diseases (NCDs) included in the analysis.

| **Metric** | **ICD-10** | **ICD-9** | **Hierarchy** |
| --- | --- | --- | --- |
| NCDs total | A46-A46·0, A66-A67·9, B18-B18·9, B33·2, B86, C00-C13·9, C15-C25·9, C30-C34·9, C37-C38·8, C40-C41·9, C43-C45·9, C47-C54·9, C56-C57·8, C58-C58·0, C60-C63·8, C64-C67·9, C68·0-C68·8, C69-C75·8, C81-C86·6, C88-C96·9, D00·1-D00·2, D01·0-D01·3, D02·0-D02·3, D03-D06·9, D07·0-D07·2, D07·4-D07·5, D09·0, D09·2-D09·3, D09·8, D10·0-D10·7, D11-D12·9, D13·0-D13·7, D14·0-D14·3, D15-D16·9, D22-D27·9, D28·0-D28·7, D29·0-D29·8, D30·0-D30·8, D31-D36, D36·1-D36·7, D37·1-D37·5, D38·0-D38·5, D39·1-D39·2, D39·8, D40·0-D40·8, D41·0-D41·8, D42-D43·9, D44·0-D44·8, D45-D47·9, D48·0-D48·6, D49·2-D49·4, D49·6, D52·1, D55-D58·9, D59·0-D59·3, D59·5-D59·6, D60-D61·9, D63·1, D64·0, D66-D67, D68·0-D69·8, D70-D75·8, D76-D78·8, D86-D86·9, D89-D89·3, E03-E07·1, E09-E11·9, E15·0, E16·0-E16·9, E20-E34·8, E36-E36·8, E65-E68, E70-E85·2, E88-E89·9, F00-F03·9, F10-F16·9, F18-F19·9, F24, F50·0-F50·5, G10-G13·8, G20-G20·9, G23-G26·0, G30-G31·9, G35-G37·9, G40-G41·9, G45-G46·8, G47·3, G61-G61·9, G70-G73·7, G90-G90·9, G93·7, G95-G95·9, G97-G97·9, H05·0-H05·1, I01-I01·9, I02·0, I05-I09·9, I11-I13·9, I20-I25·9, I27·1, I28-I28·8, I30-I31·1, I31·8-I37·8, I38-I41·9, I42·1-I42·8, I43-I43·9, I47-I48·9, I51·0-I51·4, I60-I63·9, I65-I66·9, I67·0-I67·3, I67·5-I67·7, I68·0-I68·2, I69·0-I69·3, I70·2-I70·8, I71-I73·9, I77-I89·9, I95·2-I95·3, I97-I98, I98·2, I98·9, J30-J35·9, J37-J39·9, J41-J46·9, J60-J63·8, J65-J68·9, J70-J70·9, J82, J84-J84·9, J91-J92·9, J95-J95·9, K20-K29·9, K31-K31·8, K35-K38·9, K40-K46·9, K50-K52·9, K55-K62·9, K63·5, K64-K64·9, K66·8, K67, K68-K68·9, K70-K70·3, K71·7, K74-K74·9, K75·1-K75·2, K75·4-K76·2, K76·4-K77, K77·8, K80-K83·9, K85-K86·9, K90-K91·9, K92·8, K93·8-K95·8, L00-L05·9, L08-L08·9, L10-L14·0, L51-L51·9, L88-L89·9, L93-L93·2, L97-L98·4, M00-M03·0, M03·2-M03·6, M05-M09·8, M30-M36·8, M40-M43·1, M65-M65·0, M71·0-M71·1, M72·5-M72·6, M80-M82·8, M86·3-M86·4, M87-M87·1, M88-M89·0, M89·5, M89·7-M89·9, N00-N08·8, N10-N12·9, N14-N16·8, N18-N18·9, N20-N23·0, N25-N28·1, N29-N32·0, N32·3-N32·4, N34-N34·3, N36-N36·9, N39-N39·2, N41-N41·9, N44-N44·0, N45-N45·9, N49-N49·9, N60-N60·9, N65-N65·1, N72-N72·0, N75-N77·8, N80-N81·9, N83-N83·9, N84·0-N84·1, N87-N87·9, N99-N99·9, P04·3-P04·4, P70·2, P96·0-P96·2, P96·5, Q00-Q07·9, Q10·4-Q18·9, Q20-Q28·9, Q30-Q36, Q37-Q45·9, Q50-Q87·8, Q89-Q89·8, Q90-Q93·9, Q95-Q99·8, R50·2, R78·0-R78·5, R95-R95·9, X45-X45·9, X65-X65·9, Y15-Y15·9· | 035-035·9, 036·4, 102-103·9, 133-133·6, 135-135·9, 136·6, 140-148·9, 150-158·9, 160-164·9, 170-175·9, 180-183·8, 184·0-184·4, 184·8, 185-186·9, 187·1-187·8, 188-188·9, 189·0-189·8, 190-194·8, 200-208·9, 209·0-209·1, 209·4-209·5, 210·0-210·9, 211·0-211·8, 212·0-212·8, 213-213·9, 217-220·9, 221·0-221·8, 222·0-222·8, 223·0-223·8, 224-228·9, 229·0, 229·8, 230·1-230·8, 231·0-231·2, 232-232·9, 233·0-233·2, 233·4-233·5, 233·7, 234·0-234·8, 235·0, 235·4, 235·6-235·8, 236·0-236·2, 236·4-236·5, 236·7, 237-237·3, 237·5-237·9, 238·0-238·9, 239·2-239·4, 239·6, 240-243·9, 244·0-244·1, 244·3-244·8, 245-246·9, 251-259·9, 270-273·9, 275-276, 277-277·2, 277·4-277·9, 278·0-278·8, 282-284·9, 286-286·5, 286·7-289·7, 290-292·9, 294·1-294·9, 303-303·9, 304·0-304·8, 305-305·9, 307·1, 327·2-327·8, 330-331·2, 331·5-332·0, 333-337·9, 340-341·9, 345-345·9, 349, 349·2-349·8, 353·6-353·9, 356-356·9, 357·0-357·1, 357·3-357·7, 358-359·9, 376·0-376·1, 391-391·9, 392·0, 393-398·9, 402-404·9, 410-414·9, 416·1, 417-417·9, 420-423, 423·1-423·9, 424·0-424·9, 425·0-425·3, 425·5, 425·7-425·8, 427-427·3, 427·6-427·8, 429·0, 430-435·9, 437·0-437·2, 437·4-437·8, 440·2, 440·4, 441-443·9, 446-457, 457·1-457·9, 459, 459·1-459·3, 470, 470·9-474·9, 476-476·1, 477-479, 491-493·9, 495-504·9, 506-506·9, 508-509, 515, 516-517·8, 518·6-518·7, 518·9, 519·0-519·4, 530-536·1, 536·4, 537-537·6, 537·8, 538-543·9, 550-553·6, 555-558·9, 560-560·3, 560·8-560·9, 562-562·1, 564-564·7, 565-566·9, 569·0-569·7, 571-571·9, 572·2-573·0, 573·4-577·9, 579-583·9, 585-585·9, 588-590·9, 592-593·8, 594-599·6, 599·8, 601-602·9, 604-604·9, 608·2, 610-610·9, 617-618·9, 620-620·9, 621·4-621·9, 622·1-622·7, 629-629·8, 680-689, 694-695·5, 707-707·9, 710-711·9, 714-714·3, 714·8-714·9, 730·1, 732-732·9, 733·0-733·1, 740-749·0, 749·2-758·9, 759·0-759·8, 760·7, 775·1-775·3, 779·4-779·5, 787·1, 788·0, 790·3, 798-798·0, E850, E860· | DE 1 |
| Cardiovascular diseases | B33·2, G45-G46·8, I01-I01·9, I02·0, I05-I09·9, I11-I11·9, I20-I25·9, I28-I28·8, I30-I31·1, I31·8-I37·8, I38-I41·9, I42·1-I42·8, I43-I43·9, I47-I48·9, I51·0-I51·4, I60-I63·9, I65-I66·9, I67·0-I67·3, I67·5-I67·6, I68·0-I68·2, I69·0-I69·3, I70·2-I70·8, I71-I73·9, I77-I83·9, I86-I89·0, I89·9, I98, K75·1 | 036·4, 391-391·9, 392·0, 393-398·9, 402-402·9, 410-414·9, 417-417·9, 420-423, 423·1-423·9, 424·0-424·9, 425·0-425·3, 425·5, 425·7-425·8, 427-427·3, 427·6-427·8, 429·0, 430-435·9, 437·0-437·2, 437·5-437·8, 440·2, 440·4, 441-443·9, 447-454·9, 456, 456·3-457, 457·1, 457·8-457·9, 459, 459·1-459·3 | DE 2 |
| Cardiomyopathies and myocarditis | B33·2, I40-I41·9, I42·1-I42·8, I43-I43·9, I51·4 | 422-422·9, 425·0-425·3, 425·5, 425·7-425·8, 429·0 | DE 3 |
| Alcohol cardiomyopathy | I42·6 | 425·5 | DE 4 |
| Myocarditis | B33·2, I40-I41·9, I51·4 | 422-422·9 | DE 4 |
| Endocarditis | I33-I33·9, I38-I39·9 | 421-421·9, 424·4-424·5, 424·9 | DE 3 |
| Hypertensive heart disease | I11-I11·9 | 402-402·9 | DE 3 |
| Ischaemic heart disease | I20-I25·9 | 410-414·9 | DE 3 |
| Non-rheumatic valvular heart disease | I34-I37·8 | 424·0-424·3, 424·8 | DE 3 |
| Stroke | G45-G46·8, I60-I63·9, I65-I66·9, I67·0-I67·3, I67·5-I67·6, I68·1-I68·2, I69·0-I69·3 | 430-435·9, 437·0-437·2, 437·5-437·8 | DE 3 |
| Ischaemic stroke | G45-G46·8, I63-I63·9, I65-I66·9, I67·2-I67·3, I67·5-I67·6, I69·3 | 433-435·9, 437·0-437·1, 437·5-437·8 | DE 4 |
| Intracerebral haemorrhage | I61-I62, I62·1-I62·9, I68·1-I68·2, I69·1-I69·2 | 431-432·9, 437·2 | DE 4 |
| Subarachnoid haemorrhage | I60-I60·9, I62·0, I67·0-I67·1, I69·0 | 430-430·9 | DE 4 |
| Peripheral artery disease | I70·2-I70·8, I73-I73·9 | 440·2, 440·4, 443·0-443·9 | DE 3 |
| Chronic respiratory diseases | D86-D86·2, D86·9, G47·3, J30-J35·9, J37-J39·9, J41-J46·9, J60-J63·8, J65-J68·9, J70, J70·8-J70·9, J82, J84-J84·9, J91-J92·9 | 135-135·9, 136·6, 327·2-327·8, 470, 470·9-474·9, 476-476·1, 477-479, 491-493·9, 495-504·9, 506-506·9, 508-509, 515, 516-517·8, 518·6, 518·9, 519·1-519·4 | DE 2 |
| Chronic obstructive pulmonary disease | J41-J44·9 | 491-492·9, 496-499 | DE 3 |
| Asthma | J45-J46·9 | 493-493·9 | DE 3 |
| Pneumoconiosis | J60-J63·8, J65-J65·0, J92·0 | 500-504·9 | DE 3 |
| Diabetes and kidney diseases | D63·1, E10-E11·9, I12-I13·9, N00-N08·8, N15·0, N18-N18·9, P70·2, Q61-Q62·8 | 403-404·9, 580-583·9, 585-585·9, 589-589·9, 753-753·3, 775·1 | DE 2 |
| Diabetes mellitus total | E10-E10·1, E10·3-E11·1, E11·3-E11·9, P70·2 | 775·1 | DE 3 |
| Diabetes mellitus type 1 | E10-E10·1, E10·3-E10·9, P70·2 | 775·1 | DE 4 |
| Diabetes mellitus type 2 | E11-E11·1, E11·3-E11·9 |  | DE 4 |
| Chronic kidney disease | D63·1, E10·2, E11·2, I12-I13·9, N02-N08·8, N15·0, N18-N18·9, Q61-Q62·8 | 403-404·9, 581-583·9, 585-585·9, 589-589·9, 753-753·3 | DE 3 |
| Mental disorders | F24, F50·0-F50·5 | 307·1 | DE 2 |
| Depressive disorders | NA | NA | DE 3 |
| Major depressive disorder | NA | NA | DE 4 |
| Eating disorders | F50·0-F50·5 | 307·1 | DE 3 |
| Anorexia nervosa | F50·0-F50·1 | 307·1 | DE 4 |
| Bulimia nervosa | F50·2-F50·5 | NA | DE 4 |
| Attention-deficit/hyperactive disorder | NA | NA | DE 3 |
| Autism spectrum disorders | NA | NA | DE 3 |
| Anxiety disorders | NA | NA | DE 3 |
| Bipolar disorder | NA | NA | DE 3 |
| Schizophrenia | NA | NA | DE 3 |
| Neurological disorders | F00-F03·9, G10-G13·8, G20-G20·9, G23-G24, G24·1-G25·0, G25·2-G25·3, G25·5, G25·8-G26·0, G30-G31·1, G31·8-G31·9, G35-G37·9, G40-G41·9, G61-G61·9, G70-G72, G72·2-G73·7, G90-G90·9, G95-G95·9, M33-M33·9 | 290-290·9, 294·1-294·9, 330-331·2, 331·5-332·0, 333-337·9, 340-341·9, 345-345·9, 349, 349·2-349·8, 353·6-353·9, 356-356·9, 357·0-357·1, 357·3-357·4, 357·7, 358-359·9, 775·2 | DE 2 |
| Alzheimer’s disease and other dementias | F00-F03·9, G30-G31·1, G31·8-G31·9 | 290-290·9, 294·1-294·9, 331-331·2 | DE 3 |
| Epilepsy | G40-G41·9 | 345-345·9 | DE 3 |
| Motor neuron disease | G12·2-G12·9 | 335-335·2, 335·8-335·9 | DE 3 |
| Multiple sclerosis | G35-G35·9 | 340-340·9 | DE 3 |
| Parkinson’s disease | G20-G20·9 | 332-332·0 | DE 3 |
| Musculoskeletal disorders | I27·1, I67·7, L93-L93·2, M00-M03·0, M03·2-M03·6, M05-M09·0, M09·2-M09·8, M30-M32·9, M34-M36·8, M40-M43·1, M65-M65·0, M71·0-M71·1, M80-M82·8, M86·3-M86·4, M87-M87·0, M88-M89·0, M89·5, M89·7-M89·9 | 416·1, 437·4, 446-446·9, 695·4-695·5, 710-711·9, 714-714·3, 714·8-714·9, 730·1, 732-732·9, 733·0-733·1 | DE 2 |
| Gout | NA | NA | DE 3 |
| Low back pain | NA | NA | DE 3 |
| Osteoarthritis | NA | NA | DE 3 |
| Rheumatoid arthritis | M05-M06·9, M08·0-M08·8 | 714-714·3, 714·8-714·9 | DE 3 |
| Substance use disorders | F10-F16·9, F18-F19·9, G31·2, G72·1, P04·3-P04·4, P96·1, Q86·0, R78·0-R78·5, X45-X45·9, X65-X65·9, Y15-Y15·9 | 291-292·9, 303-303·9, 304·0-304·8, 305-305·9, 357·5, 760·7, 790·3, E850, E860 | DE 2 |
| Alcohol use disorders | F10-F10·9, G31·2, G72·1, P04·3, Q86·0, R78·0, X45-X45·9, X65-X65·9, Y15-Y15·9 | 291-291·9, 303-303·9, 305·0, 357·5, 790·3, E860 | DE 3 |
| Drug use disorders | F11-F16·9, F18-F19·9, P04·4, P96·1, R78·1-R78·5 | 292-292·9, 304·0-304·8, 305, 305·1-305·9, 760·7, E850 | DE 3 |
| Cannabis use disorders | NA | NA | DE 4 |
| Cocaine use disorders | F14-F14·9, R78·2 | 304·2, 305·6 | DE 4 |
| Digestive diseases | B18-B18·9, I84-I85·9, I98·2, K20-K29·9, K31-K31·8, K35-K38·9, K40-K42·9, K44-K46·9, K50-K52·9, K55-K62, K62·2-K62·6, K62·8-K62·9, K64-K64·9, K66·8, K67, K68-K68·9, K70-K70·3, K71·7, K74-K74·9, K75·2, K75·4-K76·2, K76·4-K77, K77·8, K80-K83·9, K85-K86·9, K90-K90·9, K92·8, K93·8, M09·1 | 455-455·9, 456·0-456·2, 530-536·1, 537-537·6, 537·8, 538, 540-543·9, 550-551·1, 551·3-552·1, 552·3-553·6, 555-558·9, 560-560·3, 560·8-560·9, 562-562·1, 564-564·1, 564·5-564·7, 565-566·9, 569·1-569·5, 569·7, 571-571·9, 572·2-573·0, 573·4-577·9, 579-579·2, 579·4-579·9, 787·1 | DE 2 |
| Cirrhosis and other chronic liver diseases | B18-B18·9, I85-I85·9, I98·2, K70-K70·3, K71·7, K74-K74·9, K75·2, K75·4-K76·2, K76·4-K76·9, K77·8 | 456·0-456·2, 571-571·9, 572·2-573·0, 573·4-573·9 | DE 3 |
| Upper digestive system disorders | K21-K21·9, K22·7, K25-K29·9, K31, K31·1-K31·6, K31·8 | 530·1, 530·8, 531-535·9, 787·1 | DE 3 |
| Gynaecological diseases | D25-D26, D28·2, E28·2, N72-N72·0, N75-N77·8, N80-N81·9, N83-N83·9 | 218-219, 219·1-219·9, 236·0, 256·4, 617-618·9, 620-620·9, 621·4-621·9, 622·3-622·6, 629-629·8 | DE 3 |
| Polycystic ovarian syndrome | E28·2 | 256·4 | DE 4 |
| Neoplasms | C00-C13·9, C15-C25·9, C30-C34·9, C37-C38·8, C40-C41·9, C43-C45·9, C47-C54·9, C56-C57·8, C58-C58·0, C60-C63·8, C64-C67·9, C68·0-C68·8, C69-C75·8, C81-C86·6, C88-C96·9, D00·1-D00·2, D01·0-D01·3, D02·0-D02·3, D03-D06·9, D07·0-D07·2, D07·4-D07·5, D09·0, D09·2-D09·3, D09·8, D10·0-D10·7, D11-D12·9, D13·0-D13·7, D14·0-D14·3, D15-D16·9, D22-D24·9, D26·0-D27·9, D28·0-D28·1, D28·7, D29·0-D29·8, D30·0-D30·8, D31-D36, D36·1-D36·7, D37·1-D37·5, D38·0-D38·5, D39·1-D39·2, D39·8, D40·0-D40·8, D41·0-D41·8, D42-D43·9, D44·0-D44·8, D45-D47·9, D48·0-D48·6, D49·2-D49·4, D49·6, K62·0-K62·1, K63·5, N60-N60·9, N84·0-N84·1, N87-N87·9· | 140-148·9, 150-158·9, 160-164·9, 170-175·9, 180-183·8, 184·0-184·4, 184·8, 185-186·9, 187·1-187·8, 188-188·9, 189·0-189·8, 190-194·8, 200-208·9, 209·0-209·1, 209·4-209·5, 210·0-210·9, 211·0-211·8, 212·0-212·8, 213-213·9, 217-217·8, 219·0, 220-220·9, 221·0-221·8, 222·0-222·8, 223·0-223·8, 224-228·9, 229·0, 229·8, 230·1-230·8, 231·0-231·2, 232-232·9, 233·0-233·2, 233·4-233·5, 233·7, 234·0-234·8, 235·0, 235·4, 235·6-235·8, 236·1-236·2, 236·4-236·5, 236·7, 237-237·3, 237·5-237·9, 238·0-238·9, 239·2-239·4, 239·6, 569·0, 610-610·9, 622·1-622·2, 622·7· | DE 2 |
| Breast cancer | C50-C50·9, D05-D05·9, D24-D24·9, D48·6, D49·3 | 174-175·9, 217-217·8, 233·0, 238·3, 239·3, 610-610·9 | DE 3 |
| Cervical cancer | C53-C53·9, D06-D06·9, D26·0 | 180-180·9, 219·0, 233·1, 622·1-622·2, 622·7 | DE 3 |
| Colon and rectum cancer | C18-C21·9, D01·0-D01·3, D12-D12·9, D37·3-D37·5 | 153-154·9, 209·1, 209·5, 211·3-211·4, 230·3-230·6, 569·0 | DE 3 |
| Liver cancer | C22-C22·9, D13·4 | 155-155·9, 211·5 | DE 3 |
| Melanoma | C43-C43·9, D03-D03·9, D22-D23·9, D48·5 | 172-172·9 | DE 3 |
| Ovarian cancer | C56-C56·9, D27-D27·9, D39·1 | 183-183·0, 220-220·9, 236·2 | DE 3 |
| Prostate cancer | C61-C61·9, D07·5, D29·1, D40·0 | 185-185·9, 222·2, 236·5 | DE 3 |
| Stomach cancer | C16-C16·9, D00·2, D13·1, D37·1 | 151-151·9, 211·1, 230·2 | DE 3 |
| Uterine cancer | C54-C54·9, D07·0-D07·2, D26·1-D26·9 | 182-182·9, 233·2 | DE 3 |

ICD is the global standard for health data, clinical documentation, and statistical aggregation. Abbreviations: DE= Disease endpoint.

## Supplementary Table 4: Metadata for risk factors used.

| **Metric** | **Exposure definition** | **Theoretical minimum exposure level** | **Hierarchy** |
| --- | --- | --- | --- |
| **Metabolic risks** |  |  | RF 1 |
| High fasting plasma glucose | Serum fasting plasma glucose measured in mmol/L | 4·8–5·4 mmol/L | RF 2 |
| High systolic blood pressure | Systolic blood pressure, measured in mm Hg | 110–115 mm Hg | RF 2 |
| High body-mass index | Body-mass index, measured in kg/m² | 20–25 kg/m² | RF 2 |
| Impaired kidney function | Proportion of the population with ACR >30 mg/g or GFR <60 mL/min/1·73 m², excluding end-stage renal disease | GFR >60 mL/min/1·73 m² and ACR <30 mg/g | RF 2 |
| **Behavioural risks** |  |  | RF 1 |
| Low physical activity | Average weekly physical activity at work, home, transport-related and recreational measured by MET min per week | All adults experience 3000–4500 MET min per week | RF 2 |
| Alcohol use | Average daily alcohol consumption of pure alcohol (measured in g per day) in current drinkers who had consumed alcohol during the past 12 months | Estimated distribution 0–10 g per day | RF 2 |
| Tobacco |  |  | RF 2 |
| Smoking | Prevalence of current use of any smoked tobacco product and prevalence of former use of any smoked tobacco product; among current smokers, cigarette equivalents smoked per smoker per day and cumulative pack-years of exposure; among former smokers, number of years since quitting | All individuals are lifelong non-smokers | RF 3 |
| Second-hand smoking | Average daily exposure to air particulate matter from second-hand smoke with an aerodynamic diameter smaller than 2·5 µg, measured in µg/m³, among non-smokers | No second-hand smoke exposure | RF 3 |
| Dietary risks |  |  | RF 2 |
| Diet high in red meat | Average daily consumption of red meat (beef, pork, lamb, and goat but excluding poultry, fish, eggs, and all processed meats) | Consumption of red meat 18–27 g per day | RF 3 |
| Diet high in processed meat | Average daily consumption of meat preserved by smoking, curing, salting, or addition of chemical preservatives | Consumption of processed meat 0–4 g per day | RF 3 |
| Diet low in fruits | Average daily consumption of fruits (fresh, frozen, cooked, canned, or dried, excluding fruit juices and salted or pickled fruits) | Consumption of fruit 200–300 g  per day | RF 3 |
| Diet low in vegetables | Average daily consumption of vegetables (fresh, frozen, cooked, canned, or dried, excluding legumes and salted or pickled vegetables, juices, nuts and seeds, and starchy vegetables such as potatoes or corn) | Consumption of vegetables  290–430 g per day | RF 3 |
| Diet low in whole grains | Average daily consumption of whole grains (bran, germ, and endosperm in their natural proportion) from breakfast cereals, bread, rice, pasta, biscuits, muffins, tortillas, pancakes, and other sources | Consumption of whole grains 100–150 g per day | RF 3 |
| Diet high in sugar-sweetened beverages | Average daily consumption of beverages with ≥50 kcal per 226·8 g serving, including carbonated beverages, sodas, energy drinks, fruit drinks, but excluding 100% fruit and vegetable juices | Consumption of sugar-sweetened beverages 0–5 g per day | RF 3 |
| Diet high in sodium | 24-h urinary sodium measured in g per day | 24-h urinary sodium 1–5 g per day | RF 3 |
| Diet low in seafood omega 3 fatty acids | Average daily intake of eicosapentaenoic acid and docosahexaenoic acid | Consumption of seafood omega 3 fatty acids 200–300 mg per day | RF 3 |
| Diet low in nuts and seeds | Average daily consumption of nut and seed foods | Consumption of nuts and seeds 16–25 g per day | RF 3 |
| **Environmental and occupational risks** |  |  | RF 1 |
| Air pollution |  |  | RF 2 |
| Particulate matter pollution |  |  | RF 3 |
| Ambient particulate matter pollution | Annual average daily exposure to outdoor air concentrations of particulate matter with an aerodynamic diameter of ≤2·5 µm (PM_2·5_), measured in μg/m³ | Joint theoretical minimum risk exposure level for both household and ambient particulate matter pollution is a uniform distribution between 2·4 and 5·9 μg/m³, with burden attributed proportionally between household and particulate matter pollution on the basis of source of PM2·5 exposure in excess of theoretical minimum risk exposure level | RF 4 |
| Household air pollution from solid fuels | Individual exposure to PM_2·5_ due to use of solid cooking fuel | See ambient particulate matter pollution | RF 4 |

RF= Risk factor.

## Supplementary Table 5: This table reports descriptive information on socioeconomic indicators and care services: GDP per capita, Gini index, Hospital beds, and Doctors rate in 2002 and 2017 in the 26 Brazilian states and the Federal District.

| **State** | **GDP per capita^a^**  (R$ x 100,000) | | **Gini index^b^** | | **Hospital beds^c^**  **(**per 1,000**)** | | **Doctors^d^**  **(**per 1,000**)** | | **Primary healthcare**  (% population) | | **Private healthcare**  (% population) | |
| --- | --- | --- | --- | --- | --- | --- | --- | --- | --- | --- | --- | --- |
|  | 2002 | 2017 | 2002 | 2017 | 2002 | 2017 | 2002 | 2017 | 2002 | 2017 | 2002 | 2017 |
| **North region** |  |  |  |  |  |  |  |  |  |  |  |  |
| Acre | 2·95 | 14·21 | 0·73 | 0·68 | 2·61 | 1·65 | 0·62 | 1·11 | 42·1 | 17·2 | 3 | 5·8 |
| Amapá | 3·17 | 15·40 | 0·80 | 0·76 | 1·59 | 1·11 | 0·40 | 0·84 | 4·2 | 30 | 4·8 | 10·1 |
| Amazonas | 22·07 | 93·17 | 0·89 | 0·86 | 1·64 | 1·42 | 0·65 | 1·03 | 23·5 | 32·6 | 5·6 | 14·5 |
| Pará | 26·42 | 155·16 | 0·73 | 0·71 | 1·78 | 1·71 | 0·53 | 0·79 | 16·3 | 38·1 | 5 | 10·4 |
| Rondônia | 7·42 | 43·14 | 0·66 | 0·69 | 2·25 | 2·46 | 0·15 | 1·37 | 26·4 | 42 | 4·1 | 10·3 |
| Roraima | 2·37 | 11·99 | 0·75 | 0·73 | 2·30 | 1·67 | 0·45 | 1·35 | 29·5 | 13·7 | 2·8 | 6·4 |
| Tocantins | 5·28 | 33·93 | 0·71 | 0·72 | 2·13 | 1·67 | 0·71 | 1·35 | 24·3 | 11·9 | 3·6 | 7·3 |
| **Northeast region** |  |  |  |  |  |  |  |  |  |  |  |  |
| Alagoas | 11·52 | 52·69 | 0·71 | 0·72 | 2·33 | 1·81 | 0·97 | 1·16 | 30·6 | 11·5 | 4·8 | 12·5 |
| Bahia | 58·82 | 266·26 | 0·77 | 0·78 | 2·20 | 1·88 | 0·87 | 1·17 | 15·3 | 22·2 | 7·4 | 11·1 |
| Ceará | 28·63 | 147·59 | 0·79 | 0·78 | 2·19 | 1·85 | 0·77 | 1·11 | 45·4 | 17 | 6·7 | 14·5 |
| Maranhão | 15·87 | 89·52 | 0·73 | 0·72 | 3·69 | 1·78 | 0·46 | 0·69 | 21 | 13·2 | 3·7 | 6·8 |
| Paraíba | 12·73 | 61·83 | 0·78 | 0·78 | 3·40 | 2·05 | 0·96 | 1·34 | 23·9 | 4·5 | 6·2 | 10·8 |
| Pernambuco | 36·03 | 180·27 | 0·80 | 0·79 | 2·59 | 2·14 | 1·13 | 1·42 | 43·9 | 22·5 | 10·5 | 15·2 |
| Piauí | 7·06 | 45·35 | 0·77 | 0·76 | 2·94 | 2·13 | 0·64 | 1·09 | 35·9 | 2·1 | 3·5 | 9·5 |
| Rio Grande do Norte | 13·52 | 63·71 | 0·80 | 0·79 | 2·50 | 2·06 | 0·94 | 1·34 | 31·4 | 4·9 | 8·8 | 15·5 |
| Sergipe | 10·32 | 40·62 | 0·75 | 0·73 | 2·01 | 1·35 | 0·89 | 1·50 | 41 | 15·9 | 7·9 | 14·6 |
| **Central-west region** |  |  |  |  |  |  |  |  |  |  |  |  |
| Goiás | 38·26 | 191·42 | 0·78 | 0·78 | 4·06 | 2·59 | 1·13 | 1·64 | 30·5 | 21·8 | 9·4 | 17·1 |
| Mato Grosso | 19·00 | 125·77 | 0·71 | 0·70 | 2·61 | 2·06 | 0·73 | 1·35 | 37·1 | 32·7 | 7·5 | 17·7 |
| Mato Grosso do Sul | 16·35 | 96·01 | 0·67 | 0·68 | 2·89 | 2·02 | 1·08 | 1·79 | 37·1 | 35 | 10 | 25·3 |
| **Southeast region** |  |  |  |  |  |  |  |  |  |  |  |  |
| Espírito Santo | 26·96 | 112·67 | 0·79 | 0·74 | 2·13 | 1·94 | 1·45 | 2·14 | 19·2 | 29·4 | 21·6 | 30·4 |
| Minas Gerais | 123·28 | 575·06 | 0·82 | 0·81 | 2·71 | 1·94 | 1·46 | 2·03 | 31·8 | 18·5 | 18·5 | 25·4 |
| Rio de Janeiro | 183·91 | 668·13 | 0·84 | 0·83 | 3·12 | 2·18 | 2·93 | 2·42 | 24·1 | 31·4 | 27 | 34 |
| São Paulo | 515·16 | 2,110·09 | 0·87 | 0·87 | 2·46 | 2·07 | 2·04 | 2·47 | 18·1 | 30·6 | 37 | 41·3 |
| **South region** |  |  |  |  |  |  |  |  |  |  |  |  |
| Paraná | 87·74 | 417·58 | 0·79 | 0·77 | 3·02 | 2·44 | 1·30 | 1·93 | 26·9 | 29·5 | 15·1 | 26·6 |
| Rio Grande do Sul | 97·95 | 421·78 | 0·79 | 0·78 | 3·02 | 2·75 | 1·80 | 2·35 | 9 | 21·1 | 12·5 | 24·2 |
| Santa Catarina | 54·29 | 277·13 | 0·74 | 0·76 | 2·69 | 2·20 | 1·18 | 2·01 | 25·9 | 17·2 | 15·8 | 23·1 |

^a^GDP per capita is the state GDP in R$ divided by its population. ^b^Gini index is a measure of inequality based on household income. It ranges between 0 and 1, where 0 indicates total equality and 1 indicates total inequality. ^c^Hospital beds are calculated as the number of beds per 1,000 inhabitants. ^d^Doctors are calculated as doctors per 1,000 inhabitants.

## Supplementary Table 6. Correlations of variables used in this study.

|  | **DALYs from NCDs** | **Gini Index** | ***ln*GDP per capita** | **Bolsa Família** | **Doctors per capita** | **Coverage of private healthcare** | **Coverage of primary healthcare** | **Hospital beds** | **Percentage of people over 60 y·o** |
| --- | --- | --- | --- | --- | --- | --- | --- | --- | --- |
| **DALYs from NCDs** | 1 |  |  |  |  |  |  |  |  |
| **Gini Index** | **0·278***** | 1 |  |  |  |  |  |  |  |
| ***ln*GDP per capita** | **0·694***** | **0·531***** | 1 |  |  |  |  |  |  |
| **Bolsa Família** | **-0·134**** | **-0·127**** | **-0·208***** | 1 |  |  |  |  |  |
| **Doctors per capita** | 0·0514 | 0·0515 | 0·104* | 0·00319 | 1 |  |  |  |  |
| **Coverage of private healthcare** | **-0·099*** | -0·0952 | -0·0267 | **-0·380***** | -0·0733 | 1 |  |  |  |
| **Coverage of primary healthcare** | **0·606***** | **0·499***** | **0·822***** | **-0·424***** | **0·105*** | -0·0311 | 1 |  |  |
| **Hospital beds** | **0·553***** | **0·0994*** | **0·280***** | **-0·355***** | -0·0134 | 0·00932 | **0·246**** | 1 |  |
| **Percentage of people over 60 y·o** | **0·847***** | **0·225***** | **0·705***** | 0·0814 | **0·108*** | **-0·212***** | **0·542***** | **0·331***** | 1 |

NCDs = Non-communicable diseases; GDP = Growth domestic product; DALYs = Disability-Adjusted Life Years; Bold values express significant results. * p<0.05, ** p<0.01, *** p<0.001. Correlations reported for males.

## Supplementary Table 7: Life expectancy and health outcomes from Non-communicable diseases (NCDs) in 2002 and 2017 in the 26 Brazilian states.

| **State** | **DALYs^a^**  **(per 100,000)** | | **Life expectancy^b^**  **(years)** | |
| --- | --- | --- | --- | --- |
|  | 2002 | 2017 | 2002 | 2017 |
| **North region** |  |  |  |  |
| Acre | 16,295·40 | 15,919·04 | 73·68 | 75·89 |
| Amapá | 13,278·19 | 15,118·66 | 74·77 | 76·09 |
| Amazonas | 14,064·61 | 15,608·53 | 75·68 | 76·09 |
| Pará | 15,427·42 | 16,031·93 | 75·02 | 75·85 |
| Rondônia | 15,970·74 | 17,891·26 | 72·76 | 75·18 |
| Roraima | 14,603·41 | 15,541·41 | 72·40 | 75·16 |
| Tocantins | 16,731·22 | 17,427·64 | 74·68 | 76·36 |
| **Northeast region** |  |  |  |  |
| Alagoas | 18,770·33 | 19,097·41 | 73·09 | 74·07 |
| Bahia | 19,176·53 | 19,648·73 | 74·60 | 76·12 |
| Ceará | 18,985·50 | 17,930·08 | 75·17 | 77·22 |
| Maranhão | 18,038·12 | 16,800·83 | 74·48 | 76·54 |
| Paraíba | 19,668·23 | 19,937·70 | 74·84 | 75·71 |
| Pernambuco | 20,444·77 | 21,227·12 | 72·59 | 73·67 |
| Piauí | 18,353·01 | 18,262·71 | 75·31 | 77·17 |
| Rio Grande do Norte | 17,866·19 | 19,165·42 | 76·70 | 76·15 |
| Sergipe | 18,099·22 | 18,922·72 | 73·94 | 74·80 |
| **Central - west region** |  |  |  |  |
| Goiás | 17,271·69 | 19,023·91 | 73·91 | 75·67 |
| Mato Grosso | 16,711·28 | 17,816·10 | 73·21 | 75·93 |
| Mato Grosso do Sul | 18,339·15 | 20,077·31 | 73·25 | 75·29 |
| **Southeast region** |  |  |  |  |
| Espírito Santo | 18,733·59 | 19,590·37 | 73·26 | 76·72 |
| Minas Gerais | 19,299·56 | 20,741·40 | 74·89 | 76·67 |
| Rio de Janeiro | 22,759·10 | 23,662·02 | 70·83 | 73·88 |
| São Paulo | 20,381·50 | 21,610·79 | 72·63 | 76·13 |
| **South region** |  |  |  |  |
| Paraná | 19,885·51 | 21,606·50 | 73·03 | 75·25 |
| Rio Grande do Sul | 22,075·78 | 24,089·99 | 73·47 | 75·37 |
| Santa Catarina | 18,941·43 | 20,268·03 | 73·99 | 76·66 |

^a^DALYs are calculated by the sum of YLL and YLD (one DALY equals one lost year of healthy life); ^b^Life expectancy is shown for the age group of 1-4 years old; YLL=year of life lost; YLD=years lived with disability; DALYs=disability-adjusted life years.

## Supplementary Table 8: Fixed effect regression coefficients of the Gini index for disability-adjusted life years (DALYs per 100,000) of non-communicable diseases (NCDs) in the 26 Brazilian states. The time period spans from 2002 to 2017.

| **Outcome** | Men | Women |
| --- | --- | --- |
| **NCDs total** | 4,605  (-1,740 - 10,950) | 404,1  (-6,496 - 7,304) |
| **Cardiovascular diseases** | 3,137*  (438·4 - 5,836) | 1,446  (-839·2 - 3,731) |
| Cardiomyopathies and myocarditis | 28·55  (-242·5 - 299·5) | -171·6  (-486·6 - 143·4) |
| Alcohol cardiomyopathy | 15·62  (-76·61 - 107·8) | -5·11  (-25·23 - 14·99) |
| Myocarditis | -10·60  (-31·18 - 9·98) | -9·92  (-26·41 - 6·55) |
| Endocarditis | -5·43  (-53·81 - 42·93) | -6·71  (-41·11 - 27·67) |
| Hypertensive heart disease | 219·2  (-204·1 - 642·5) | 223·6  (-161·0 - 608·3) |
| Ischaemic heart disease | 2,300**  (779·4 - 3,821) | 1,074*  (228·6 - 1,918) |
| Non-rheumatic valvular heart disease | 30·33  (-39·17 - 99·83) | 24·22  (-7·06 - 18·15) |
| Stroke | 476,5  (-931·7 - 1,885) | 339·0  (-977·9 - 1,656) |
| Ischaemic stroke | 123·5  (-344·4 - 591·4) | 127·0  (-203·0 - 457·0) |
| Intracerebral haemorrhage | 324·9  (-488·2 - 1,138) | 122·8  (-631·9 - 877·4) |
| Subarachnoid haemorrhage | 28·16  (-139·0 - 195·3) | 89·16  (-174·9 - 353·2) |
| Peripheral artery disease | -5·22  (-20·35 - 9·90) | -8·84  (-25·54 - 7·85) |
| **Chronic respiratory diseases** | 346·1  (-348·5 - 1,041) | 94·55  (-608·4 - 797·5) |
| Chronic obstructive pulmonary disease | 408·9  (-186·0 - 1,004) | 209·0  (-186·4 - 604·4) |
| Asthma | -93·33  (-281·6 - 94·88) | -83·64  (-380·2 - 212·9) |
| Pneumoconiosis | 8·00  (-0·27 - 16·29) | -4·71  (-14·77 - 5·34) |
| **Diabetes and kidney diseases** | 1,199**  (341·3 - 2,057) | **1,009‡**  **(345·0 - 1,672)** |
| Diabetes mellitus total | 893·3*  (127·7 - 1,659) | **706·2†**  **(120·5 - 1,292)** |
| Diabetes mellitus type 1 | 189·0  (-8·872 - 386·9) | 12·59  (-145·6 - 170·8) |
| Diabetes mellitus type 2 | 704·3*  (106·8 - 1,302) | **693·6†**  **(159·2 - 1,228)** |
| Chronic kidney disease | 310·2**  (99·41 - 520·9) | 314,5*  (44·66 - 584·4) |
| **Mental disorders** | -91·88  (-369·2 - 185·5) | -221·2  (-905·3 - 463·0) |
| Depressive disorders | -33·04  (-294·1 - 228·0) | -213·7  (-829·8 - 402·3) |
| Major depressive disorder | -26·68  (-289·0 - 235·7) | -202·8  (-820·3 - 414·6) |
| Eating disorders | -7·21*  (-14·27 - -0·16) | -7·58  (-22·67 - 7·495) |
| Anorexia nervosa | -0·55  (-1·81 - 0·70) | -1·77  (-5·67 - 2·12) |
| Bulimia nervosa | -6·66*  (-12·80 - -0·52) | -5·81  (-17·24 - 5·61) |
| Attention-deficit/hyperactive disorder | **-4·01†**  **(-7·47 - -0·54)** | **-1·58†**  **(-2·88 - -0·28)** |
| Autism spectrum disorders | **-2·42†**  **(-4·32 - -0·53)** | -0·81  (-1·95 - 0·32) |
| Anxiety disorders | -6·61  (-41·25 - 28·02) | 11·49  (-87·43 - 110·4) |
| Bipolar disorder | -9·87  (-43·23 - 23·49) | -3·52  (-40·77 - 33·72) |
| Schizophrenia | 14·21  (-4·44 - 32·87) | 15·27  (-6·20 - 36·75) |
| **Neurological disorders** | -92·83  (-485·6 - 299·9) | -157·9  (-551·5 - 235·7) |
| Alzheimer’s disease and other dementias | -104·2  (-331·5 - 123·0) | -103·5  (-304·9 - 97·91) |
| Epilepsy | 28·16  (-118·4 - 174·8) | -31·21  (-115·0 - 52·56) |
| Motor neuron disease | 6·63  (-9·29 - 22·57) | -4·30  (-15·94 - 7·32) |
| Multiple sclerosis | 1·08  (-2·64 - 4·81) | **5·38‡**  **(2·24 - 8·52)** |
| Parkinson’s disease | 4·76  (-25·53 - 35·05) | 7·14  (-7·84 - 22·14) |
| **Musculoskeletal disorders** | 155·3  (-53·86 - 364·4) | 215·1  (-42·65 - 472·8) |
| Gout | 2·17*  (0·38 - 3·96) | 0·78  (-0·07 - 1·65) |
| Low back pain | 60·50  (-82·86 - 203·9) | 50·64  (-142·1 - 243·4) |
| Osteoarthritis | 13·67*  (1·06 - 26·28) | 11·77**  (3·07 - 20·46) |
| Rheumatoid arthritis | -2·96  (-15·73 - 9·78) | 5·54  (-7·06 - 18·15) |
| **Substance use disorders** | 797·8  (-20·10 - 1,616) | 88·29**  (25·39 - 151·2) |
| Alcohol abuse | 923·4*  (217·6 - 1,629) | **57·33‡**  **(20·45 - 94·21)** |
| Drug use disorders | -125·6  (-480·8 - 229·5) | 30·96  (-6·631 - 68·56) |
| Cannabis use disorders | 1·66  (-0·79 - 4·13) | 1·70  (-0·28 - 3·69) |
| Cocaine use disorders | -36·52  (-84·04 - 11·00) | 3·72  (-2·33 - 9·77) |
| **Digestive diseases** | 1,080  (-74·06 - 2,234) | 255·3  (-106·4 - 617·1) |
| Cirrhosis and other chronic liver diseases | 1,020*  (105·9 - 1,934) | 207·5  (-5·52 - 420·6) |
| Alcoholic Cirrhosis | 269·5*  (15·69 - 523·3) | **39·48†**  **(4·50 - 74·46)** |
| Non-alcoholic steatohepatitis (NASH) | **263·2‡**  **(104·7 - 421·6)** | **78·86†**  **(12·46 - 145·3)** |
| Upper digestive system disorders | 9·14  (-163·5 - 181·8) | -5·04  (-141·7 - 131·7) |
| **Gynecological diseases** | NA | 75·89  (-15·25 - 167·0) |
| Polycystic ovarian syndrome | NA | 2·51  (-0·59 - 5·61) |
| **Neoplasms** | 332·9  (-1,119 - 1,785) | -42·95  (-1,151 - 1,065) |
| Breast cancer | 2·08  (-6·19 - 10·36) | 107·3  (-355·5 - 570·1) |
| Cervical cancer | NA | -60·34  (-314·4 - 193·7) |
| Colon and rectum cancer | 47·35  (-32·04 - 126·7) | -37·03  (-129·5 - 55·43) |
| Liver cancer | -41·14  (-163·5 - 81·22) | -5·34  (-47·82 - 37·13) |
| Melanoma | 10·23  (-14·66 - 35·13) | -2·85  (-15·46 - 9·75) |
| Ovarian cancer | NA | 37·75  (-26·79 - 102·3) |
| Prostate cancer | -6·14  (-265·2 - 252·9) | NA |
| Stomach cancer | 29·78  (-206·5 - 266·1) | 56·97  (-24·89 - 138·8) |
| Uterine cancer | NA | -7·89  (-33·63 - 17·84) |

NCDs were regressed one at the time. Coefficients are reported x 10^-3^. Bold values express significant results in both unadjusted and adjusted models. Significant values that are not in bold indicate significances only in the adjusted model; * or † p<0·05, ** or ‡ p<0·01. Variables included in the adjusted model: doctors per 1,000 habitants, hospital beds per 1,000 habitants, coverage of private healthcare, coverage of primary care, Bolsa Família transfer, and percentage of the population over 60 years of age; NA= not applicable.

## Supplementary Table 9: Fixed effect regression coefficients of the Gini index for years of life lost (YLLs per 100,000) of non-communicable diseases (NCDs) in 26 Brazilian states. The time period spans from 2002 to 2017.

| **Outcome** | Men | Women |
| --- | --- | --- |
| **NCDs total** | 4,109  (-1,754 - 9,973) | -188·0  (-6,393 - 6,017) |
| **Cardiovascular diseases** | 3,090*  (424·1 - 5,755) | 1,408  (-847·0 - 3,662) |
| Cardiomyopathies and myocarditis | 25·04  (-248·2 - 298·2) | -176·0  (-492·9 - 140·9) |
| Alcohol cardiomyopathy | 15·05  (-77·54 - 107·6) | -5·39  (-26·02 - 15·22) |
| Myocarditis | -10·74  (-31·36 - 9·88) | -10·02  (-26·51 - 6·47) |
| Endocarditis | -5·46  (-53·75 - 42·82) | -6·71  (-41·07 - 27·63) |
| Hypertensive heart disease | 214·6  (-206·3 - 635·4) | 215·8  (-167·8 - 599·3) |
| Ischaemic heart disease | 1,069*  (226·9 - 1,912) | 2,284**  (773·6 - 3,795) |
| Non-rheumatic valvular heart disease | 29·56  (-39·98 - 99·11) | 22·53  (-43·65 - 88·72) |
| Stroke | 315·1  (-984·0 - 1,614) | 453·8  (-942·9 - 1,851) |
| Ischaemic stroke | 105·4  (-355·6 - 566·4) | 109·2  (-213·7 - 432·1) |
| Intracerebral haemorrhage | 321·9  (-486·7 - 1,130) | 119·0  (-626·2 - 864·2) |
| Subarachnoid haemorrhage | 26·56  (-140·5 - 193·6) | 86·88  (-175·9 - 349·7) |
| Peripheral artery disease | -5·50  (-20·61 - 9·60) | -9·256  (-25·90 - 7·38) |
| **Chronic respiratory diseases** | 362·4  (-203·5 - 928·2) | 50·31  (-500·7 - 601·3) |
| Chronic obstructive pulmonary disease | 352·4  (-180·8 - 885·6) | 153·9  (-182·2 - 490·1) |
| Asthma | -15·53  (-86·52 - 55·46) | -68·31  (-262·4 - 125·7) |
| Pneumoconiosis | 8·10  (-0·19 - 16·41) | -4·53  (-14·53 - 5·45) |
| **Diabetes and kidney diseases** | 1,041**  (277·2 - 1,804) | **762·2‡**  **(227·1 - 1,297)** |
| Diabetes mellitus total | 755·7*  (71·03 - 1,440) | **535·7†**  **(40·70 - 1,031)** |
| Diabetes mellitus type 1 | 189·0  (-9·964 - 388·0) | 11·70  (-147·1 - 170·5) |
| Diabetes mellitus type 2 | 566·7*  (56·17 - 1,077) | **524·0†**  **(81·42 - 966·6)** |
| Chronic kidney disease | 289·6**  (90·46 - 488·8) | 238·4  (-15·15 - 492·0) |
| **Mental disorders** | 0·15  (-0·20 - 0·50) | 0·38  (-0·28 - 1·05) |
| Depressive disorders # | N/A | N/A |
| Major depressive disorder # | N/A | N/A |
| Eating disorders | 0·15  (-0·20 - 0·50) | 0·38  (-0·28 - 1·05) |
| Anorexia nervosa | 0·06  (-0·08 - 0·21) | 0·37  (-0·18 - 0·94) |
| Bulimia nervosa | 0·08  (-0·16 - 0·32) | 0·004  (-0·25 - 0·26) |
| Attention-deficit/hyperactive disorder # | NA | NA |
| Autism spectrum disorders # | NA | NA |
| Anxiety disorders # | NA | NA |
| Bipolar disorders # | NA | NA |
| Schizophrenia # | NA | NA |
| **Neurological disorders** | -7·48  (-258·7 - 243·7) | -111·8  (-342·1 - 118·5) |
| Alzheimer’s disease and other dementias | -94·98  (-280·6 - 90·67) | -82·69  (-266·2 - 100·8) |
| Epilepsy | 59·73  (-21·87 - 141·3) | -12·72  (-64·99 - 39·56) |
| Motor neuron disease | 6·63  (-9·19 - 22·46) | -4·25  (-15·81 - 7·29) |
| Multiple sclerosis | 1·42  (-1·05 - 3·90) | 2·15  (-1·06 - 5·38) |
| Parkinson’s disease | 3·11  (-25·95 - 32·17) | 6·67  (-8·34 - 21·69) |
| **Musculoskeletal disorders** | 16·06  (-10·57 - 42·70) | 4·05  (-62·30 - 70·41) |
| Gout # | NA | NA |
| Low back pain # | NA | NA |
| Osteoarthritis # | NA | NA |
| Rheumatoid arthritis | -0·33  (-3·38 - 2·71) | -1·45  (-8·85 - 5·94) |
| **Substance use disorders** | 588·5  (-88·93 - 1,266) | 52·19**  (17·18 - 87·21) |
| Alcohol abuse | 725·3*  (113·8 - 1,337) | **32·08‡**  **(3·91 - 60·25)** |
| Drug use disorders | -136·8  (-403·2 - 129·5) | 20·11*  (0·56 - 39·65) |
| Cannabis use disorders # | N/A | N/A |
| Cocaine use disorders | -32·81  (-69·43 - 3·81) | 1·80  (-4·68 - 8·28) |
| **Digestive diseases** | 1,070  (-33·34 - 2,173) | 242·2  (-49·50 - 533·9) |
| Cirrhosis and other chronic liver diseases | 1,008*  (96·07 - 1,921) | 207·1  (-4·48 - 418·7) |
| Alcoholic Cirrhosis | 268·4*  (16·88 - 519·9) | **38·88†**  **(4·11 - 73·65)** |
| Non-alcoholic steatohepatitis (NASH) | **258·7‡**  **(101·1 - 416·2)** | **77·89†**  **(12·82 - 143·0)** |
| Upper digestive system disorders | 55·55  (-36·26 - 147·4) | 19·18  (-4·15 - 42·51) |
| **Gynecological diseases** | NA | 2·98  (-1·07 - 7·05) |
| Polycystic ovarian syndrome | NA | 0·35  (-0·22 - 0·93) |
| **Neoplasms** | 327·9  (-1,111 - 1,767) | -43·85  (-1,130 - 1,042) |
| Breast cancer | 2·02  (-5·84 - 9·90) | 103·2  (-347·4 - 553·8) |
| Cervical cancer | NA | -59·23  (-305·2 - 186·7) |
| Colon and rectum cancer | 47·19  (-29·05 - 123·4) | -34·68  (-126·5 - 57·11) |
| Liver cancer | -40·61  (-161·9 - 80·68) | -5·44  (-47·59 - 36·71) |
| Melanoma | 10·17  (-13·54 - 33·87) | -2·49  (-14·16 - 9·16) |
| Ovarian cancer | NA | 36·50  (-26·12 - 99·12) |
| Prostate cancer | -8·27  (-256·7 - 240·1) | NA |
| Stomach cancer | 29·80  (-204·2 - 263·8) | 56·46  (-24·50 - 137·4) |
| Uterine cancer | NA | -7·09  (-31·36 - 17·16) |

NCDs were regressed one at a time. Coefficients are reported x 10^-3^. Bold values express significant results in both unadjusted and adjusted models. Significant values that are not in bold indicate significances only in the adjusted model; * or † p<0·05, ** or ‡ p<0·01. Variables included in the adjusted model: doctors per 1,000 habitants, hospital beds per 1,000 habitants, coverage of private healthcare, coverage of primary care, Bolsa Família transfer, and percentage of the population over 60 years of age; NA= not applicable.

## Supplementary Table 10: Fixed effect regression coefficients of the Gini index for risk factors of non-communicable diseases (NCDs) in the 26 Brazilian states. Risk factors are regressed one at the time. Coefficients are reported x 10^-3^. The time period spans from 2002 to 2017.

| **Risk Factor** | Men | Women |
| --- | --- | --- |
| **Metabolic risks** |  |  |
| High fasting plasma glucose | 1·87  (-0·05 - 3·80) | -0·13  (-2·17 - 1·90) |
| High systolic blood pressure | 0·02  (-1·43 - 1·47) | 1·58*  (0·04 - 3·12) |
| High Body-Mass Index | -1·66  (-8·33 - 5·00) | -3·69  (-11·39 - 4·00) |
| Impaired kidney function | 0·22  (-0·02 - 0·46) | 0·30*  (0·05 - 0·54) |
| **Behavioural risks** |  |  |
| Low physical activity | 0·0001 (-0·01 - 0·01 | 0·001 (-0·02 - 0·02) |
| Alcohol use | **5·53‡**  **(1·75 - 9·31)** | 0·75  (-0·11 - 1·62) |
| Smoking | -0·56 (-2·72 - 1·60) | 2·64 (-0·64 - 5·93) |
| Second-hand smoking | 5·97 (-19·27 - 31·21) | 7·54 (-14·15 - 29·24) |
| **Dietary risks** |  |  |
| Diet high in red meat | -1·09  (-6·75 - 4·56) | **-9·45‡**  **(-15·24 - -3·67)** |
| Diet high in processed meat | 0·55  (-1·53 - 2·63) | 0·27  (-0·92 - 1·48) |
| Diet low in fruits | 1·80  (-3·11 - 6·71) | 0·81  (-4·79 - 6·42) |
| Diet high in sodium | 0·19  (-0·62 - 1·02) | 0·51  (-0·14 - 1·16) |
| Diet high in sugar-sweetened beverages | -3·82*  (-7·07 - -0·58) | -2·92**  (-4·54 - -1·29) |
| Diet low in vegetables | **7·26‡**  **(2·65 - 11·87)** | **6·00‡**  **(1·94 - 10·06)** |
| Diet low in whole grains | 1·19*  (0·21 - 2·18) | 1·93**  (1·14 - 2·73) |
| Diet low in nuts and seeds | 2·39**  (1·03 - 3·74) | 2·51**  (1·34 - 3·69) |

Bold values express significant results in both unadjusted and adjusted models. Significant values that are not in bold indicate significances only in the adjusted model; * or † p<0·05, ** or ‡ p<0·01. Variables included in the adjusted model: doctors per 1,000 habitants, hospital beds per 1,000 habitants, coverage of private healthcare, coverage of primary care, Bolsa Família transfer, and percentage of the population over 60 years of age.


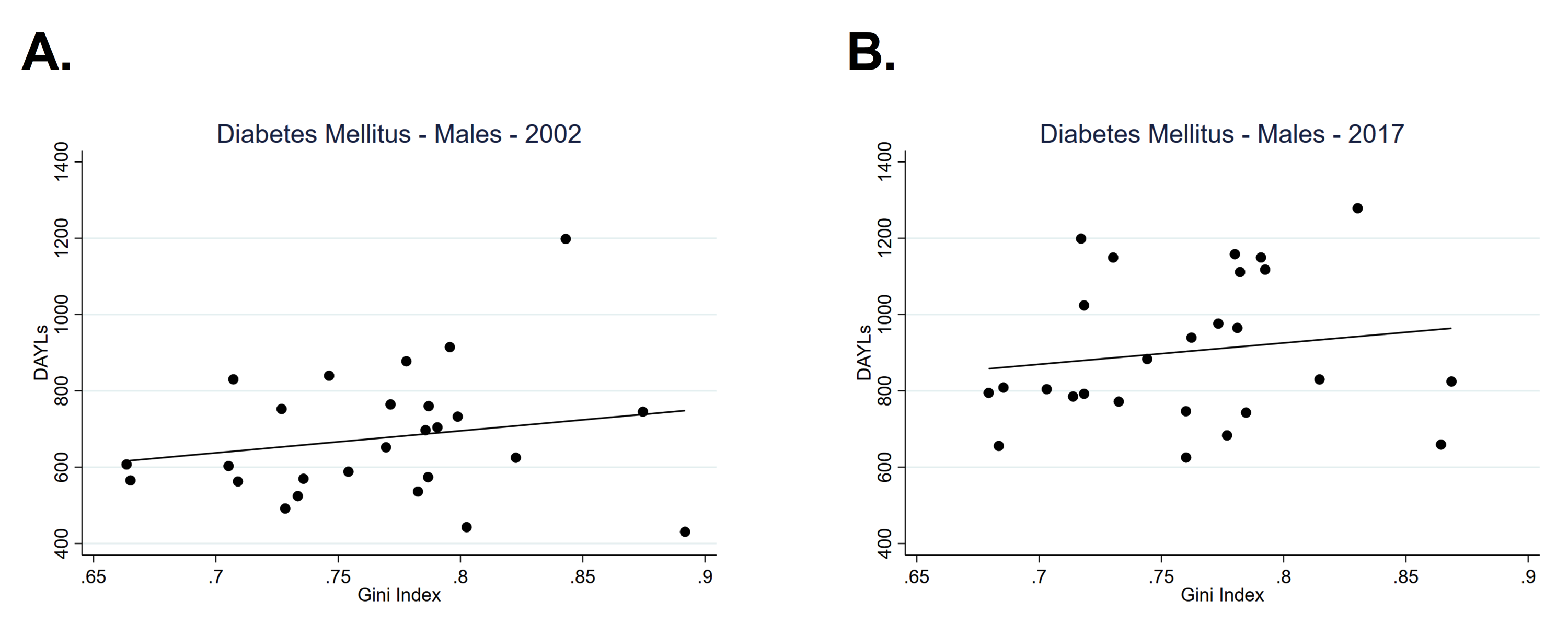


**Supplementary Figure 1. Positive linear correlation between DALYs of diabetes mellitus in men and Gini Index in 2002 (A) and 2017 (B).** DALYs is expressed as DALYs per 100,000 people.


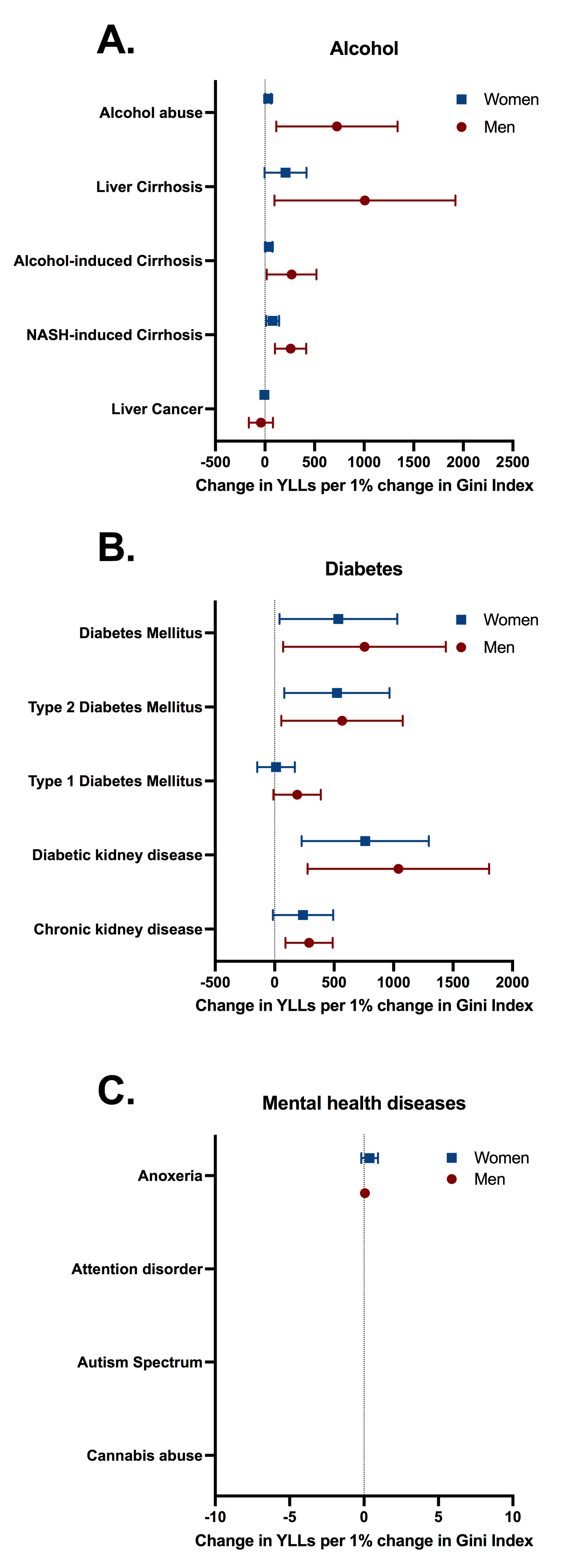


**Supplementary Figure 2. Associations between Gini Index and years of life lost (YLLs) rate of (A) alcohol-, (B) diabetes-, and (C) mental health-related diseases in men and women in Brazil.** Variables included in the model: doctors per 1,000 habitants, hospital beds per 1,000 habitants, coverage of private healthcare, coverage of primary care, Bolsa Família transfer, percentage of the population over 60 years of age and state and year fixed effects. Attention disorder, autism spectrum and cannabis use are not formally considered causes of death, therefore there is no YLLs data available for these diseases.


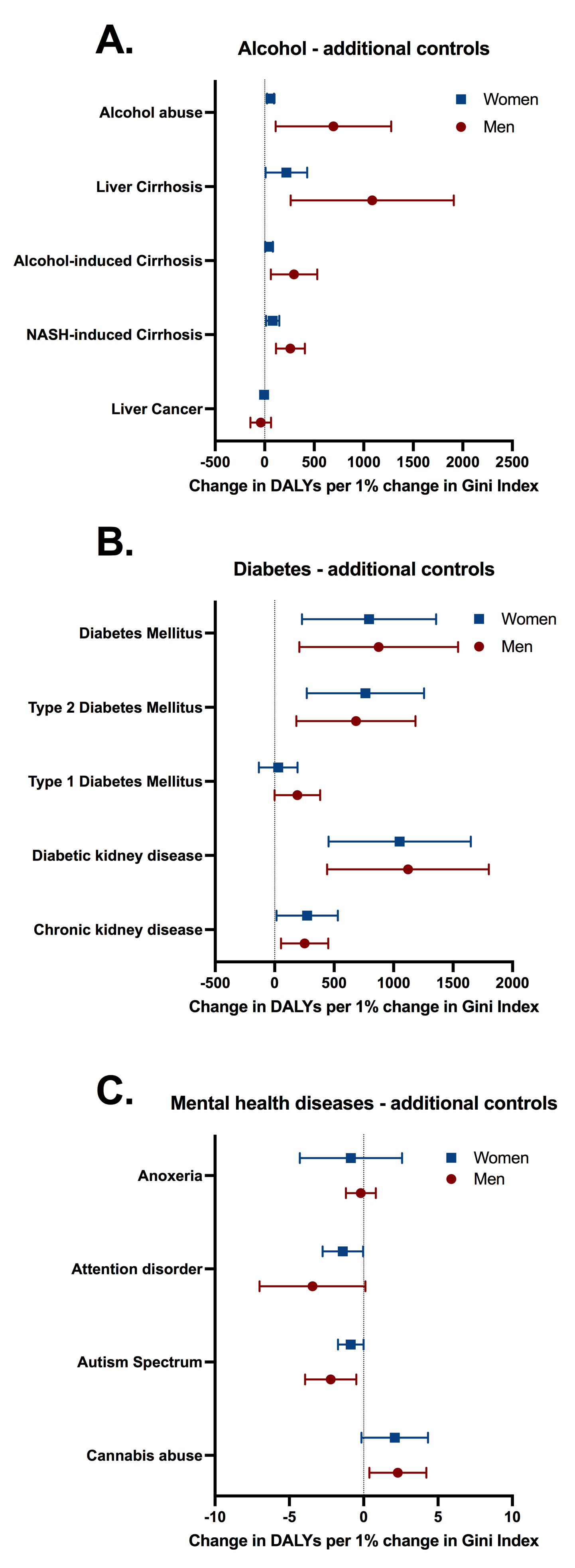


**Supplementary Figure 3. Robustness test: Associations between Gini Index and DALYs rate for (A) alcohol-, (B) diabetes-, and (C) mental health-related diseases in men and women in Brazil using additional control variables.** Variables included in the model: doctors per 1,000 habitants, hospital beds per 1,000 habitants, coverage of private healthcare, coverage of primary care, Bolsa Família transfer, percentage of the population over 60 years of age, percentage of urban population, percentage of white individuals and state and year fixed effects.
