# Supplementary material for: Income inequality and non-communicable disease mortality and morbidity in Brazil States: a longitudinal analysis 2002-2017
Source: Lancet Reg Health Am. 2021 Aug 17;2:100042. doi: 10.1016/j.lana.2021.100042 (PMC9904117; doi:10.1016/j.lana.2021.100042)
Supplement: Supplementary file 3 [file mmc3.docx]

**Supplement to:** Gaspar RS*, Rossi L, Hone T, Dornelles AZ. **Income inequality and non-communicable disease mortality and morbidity in Brazil States: a longitudinal analysis 2002-2017**

*Corresponding author:

Renato Simões Gaspar

E-mail: renatosgaspar@gmail.com

Tel: +44 11 8378 7047

## Supplementary Table 1: Descriptive information on variables and data sources explored across 26 Brazilian states from 2002 to 2017.

^a^Gini Index was not measured in 2002 and 2017; ^b^Hospital beds were not available in 2004.

## Supplementary Table 2: Glossary of metrics.

## Supplementary Table 3: International Classification of Diseases (ICD) codes and hierarchy for non-communicable diseases (NCDs) included in the analysis.

ICD is the global standard for health data, clinical documentation, and statistical aggregation. Abbreviations: DE= Disease endpoint.

## Supplementary Table 4: Metadata for risk factors used.

RF= Risk factor.

## Supplementary Table 5: This table reports descriptive information on socioeconomic indicators and care services: GDP per capita, Gini index, Hospital beds, and Doctors rate in 2002 and 2017 in the 26 Brazilian states and the Federal District.

^a^GDP per capita is the state GDP in R$ divided by its population. ^b^Gini index is a measure of inequality based on household income. It ranges between 0 and 1, where 0 indicates total equality and 1 indicates total inequality. ^c^Hospital beds are calculated as the number of beds per 1,000 inhabitants. ^d^Doctors are calculated as doctors per 1,000 inhabitants.

## Supplementary Table 6. Correlations of variables used in this study.

NCDs = Non-communicable diseases; GDP = Growth domestic product; DALYs = Disability-Adjusted Life Years; Bold values express significant results. * p<0.05, ** p<0.01, *** p<0.001. Correlations reported for males.

## Supplementary Table 7: Life expectancy and health outcomes from Non-communicable diseases (NCDs) in 2002 and 2017 in the 26 Brazilian states.

^a^DALYs are calculated by the sum of YLL and YLD (one DALY equals one lost year of healthy life); ^b^Life expectancy is shown for the age group of 1-4 years old; YLL=year of life lost; YLD=years lived with disability; DALYs=disability-adjusted life years.

## Supplementary Table 8: Fixed effect regression coefficients of the Gini index for disability-adjusted life years (DALYs per 100,000) of non-communicable diseases (NCDs) in the 26 Brazilian states. The time period spans from 2002 to 2017.

NCDs were regressed one at the time. Coefficients are reported x 10^-3^. Bold values express significant results in both unadjusted and adjusted models. Significant values that are not in bold indicate significances only in the adjusted model; * or † p<0·05, ** or ‡ p<0·01. Variables included in the adjusted model: doctors per 1,000 habitants, hospital beds per 1,000 habitants, coverage of private healthcare, coverage of primary care, Bolsa Família transfer, and percentage of the population over 60 years of age; NA= not applicable.

## Supplementary Table 9: Fixed effect regression coefficients of the Gini index for years of life lost (YLLs per 100,000) of non-communicable diseases (NCDs) in 26 Brazilian states. The time period spans from 2002 to 2017.

NCDs were regressed one at a time. Coefficients are reported x 10^-3^. Bold values express significant results in both unadjusted and adjusted models. Significant values that are not in bold indicate significances only in the adjusted model; * or † p<0·05, ** or ‡ p<0·01. Variables included in the adjusted model: doctors per 1,000 habitants, hospital beds per 1,000 habitants, coverage of private healthcare, coverage of primary care, Bolsa Família transfer, and percentage of the population over 60 years of age; NA= not applicable.

## Supplementary Table 10: Fixed effect regression coefficients of the Gini index for risk factors of non-communicable diseases (NCDs) in the 26 Brazilian states. Risk factors are regressed one at the time. Coefficients are reported x 10^-3^. The time period spans from 2002 to 2017.

Bold values express significant results in both unadjusted and adjusted models. Significant values that are not in bold indicate significances only in the adjusted model; * or † p<0·05, ** or ‡ p<0·01. Variables included in the adjusted model: doctors per 1,000 habitants, hospital beds per 1,000 habitants, coverage of private healthcare, coverage of primary care, Bolsa Família transfer, and percentage of the population over 60 years of age.

**Supplementary Figure 1. Positive linear correlation between DALYs of diabetes mellitus in men and Gini Index in 2002 (A) and 2017 (B).** DALYs is expressed as DALYs per 100,000 people.

**Supplementary Figure 2. Associations between Gini Index and years of life lost (YLLs) rate of (A) alcohol-, (B) diabetes-, and (C) mental health-related diseases in men and women in Brazil.** Variables included in the model: doctors per 1,000 habitants, hospital beds per 1,000 habitants, coverage of private healthcare, coverage of primary care, Bolsa Família transfer, percentage of the population over 60 years of age and state and year fixed effects. Attention disorder, autism spectrum and cannabis use are not formally considered causes of death, therefore there is no YLLs data available for these diseases.

**Supplementary Figure 3. Robustness test: Associations between Gini Index and DALYs rate for (A) alcohol-, (B) diabetes-, and (C) mental health-related diseases in men and women in Brazil using additional control variables.** Variables included in the model: doctors per 1,000 habitants, hospital beds per 1,000 habitants, coverage of private healthcare, coverage of primary care, Bolsa Família transfer, percentage of the population over 60 years of age, percentage of urban population, percentage of white individuals and state and year fixed effects.
