# Supplementary material for: Income inequality and non-communicable disease mortality and morbidity in Brazil States: a longitudinal analysis 2002-2017
Source: Lancet Reg Health Am. 2021 Aug 17;2:100042. doi: 10.1016/j.lana.2021.100042 (PMC9904117; doi:10.1016/j.lana.2021.100042)
Supplement: Supplementary file 6 [file mmc6.pdf]

Checklist of information that should be included in new reports of global health estimates

Item #	Checklist item	Reported on page #
**Objectives and funding**		
**1**	Define the indicator(s), populations (including age, sex, and geographic entities), and time period(s) for which estimates were made.	6-8
**2**	List the funding sources for the work.	2, 8
**Data Inputs**		
For all data inputs from multiple sources that are synthesized as part of the study:		
**3**	Describe how the data were identified and how the data were accessed.	6
**4**	Specify the inclusion and exclusion criteria. Identify all ad-hoc exclusions.	6
**5**	Provide information on all included data sources and their main characteristics. For each data source used, report reference information or contact name/institution, population represented, data collection method, year(s) of data collection, sex and age range, diagnostic criteria or measurement method, and sample size, as relevant.	6 and Suppl Table 1
**6**	Identify and describe any categories of input data that have potentially important biases (e.g., based on characteristics listed in item 5).	N/A
For data inputs that contribute to the analysis but were not synthesized as part of the study:		
**7**	Describe and give sources for any other data inputs.	N/A
For all data inputs:		
**8**	Provide all data inputs in a file format from which data can be efficiently extracted (e.g., a spreadsheet rather than a PDF), including all relevant meta-data listed in item 5. For any data inputs that cannot be shared because of ethical or legal reasons, such as third-party ownership, provide a contact name or the name of the institution that retains the right to the data.	Supporting Information
**Data analysis**		
**9**	Provide a conceptual overview of the data analysis method. A diagram may be helpful.	6 - 8
**10**	Provide a detailed description of all steps of the analysis, including mathematical formulae. This description should cover, as relevant, data cleaning, data pre-processing, data adjustments and weighting of data sources, and mathematical or statistical model(s).	7 - 8
**11**	Describe how candidate models were evaluated and how the final model(s) were selected.	7 - 8
**12**	Provide the results of an evaluation of model performance, if done, as well as the results of any relevant sensitivity analysis.	7 - 8
**13**	Describe methods for calculating uncertainty of the estimates. State which sources of uncertainty were, and were not, accounted for in the uncertainty analysis.	6 - 8
**14**	State how analytic or statistical source code used to generate estimates can be accessed.	7 - 8
**Results and Discussion**		
**15**	Provide published estimates in a file format from which data can be efficiently extracted.	Suppl Material
**16**	Report a quantitative measure of the uncertainty of the estimates (e.g. uncertainty intervals).	9 - 10
**17**	Interpret results in light of existing evidence. If updating a previous set of estimates, describe the reasons for changes in estimates.	11 - 14
**18**	Discuss limitations of the estimates. Include a discussion of any modelling assumptions or data limitations that affect interpretation of the estimates.	13 - 14

This checklist should be used in conjunction with the GATHER statement and Explanation and Elaboration document, found on gather-statement.org
